# Optical Smart-Wheel Sensing of Tire Deformation for Tire State and Load Estimation

**DOI:** 10.3390/s26196284

**Published:** 2026-10-03

**Authors:** Halil Yahya Yeşilyurt, Özgen Akalın

**Affiliations:** Mechanical Engineering Department, Istanbul Technical University, Gumussuyu, İstanbul 34437, Türkiye; akalin@itu.edu.tr

**Keywords:** smart wheel, intelligent tire, optical sensing, tire deformation, tire–road interaction, slip angle, image processing, vehicle dynamics

## Abstract

Tire–road interaction is increasingly important for situational awareness in autonomous vehicles and advanced driver-assistance systems, as tire deformation contains information that is related to both vehicle kinematics and tire forces. Optical sensing offers a direct means of observing this interaction with minimal influence on tire dynamics. In this study, an optical smart-wheel prototype was developed using a single high-speed camera rotating with the wheel and observing a circular-marker grid applied to the tire’s inner liner. Camera calibration and distance-dependent imaging tests were used to support geometric correction and pixel-to-length conversion, after which the system was evaluated using static and on-road dynamic tests. Static drum calibration established a relationship between the diagonal-distance metric and vertical wheel load (R^2^ = 0.9992; RMSE = 33.37 N). Initial vehicle-level multi-point validation over 30 static conditions and loads up to approximately 3.92 kN yielded a prediction R^2^ of 0.9933 and an RMSE of 91.05 N. Dynamic tests revealed increasing longitudinal and lateral deformation with increasing maneuver speed and decreasing inflation pressure, together with direction-dependent lateral responses consistent with wheel-load transfer. Across the cases included in the cycle-variation analysis, the coefficients of variation ranged from 1.88% to 5.10% for longitudinal amplitude and from 6.08% to 19.98% for lateral peak deformation. These results demonstrate sensitivity to operating conditions and indicate a potential association between lateral deformation and slip angle, rather than constituting direct tire-force or slip-angle estimation. The proposed metric-based approach provides a promising lower-complexity alternative to full-field stereo-based deformation measurement for future real-time tire-state sensing and investigation of force-related deformation indicators.

## 1. Introduction

As mechatronics has expanded its presence in the automotive sector—along with the refinement of internal combustion engines, the electrification of vehicles, and the widespread adoption of various driver-assistance systems on the path toward autonomous vehicles—this has also accelerated the transformation of vehicles into increasingly “smart” industrial products. Given that autonomous vehicles are expected to become increasingly widespread in the coming years, the growing need for smarter vehicles is also becoming evident. In this context, measuring or estimating tire forces and moments—which represent the dynamics of the interaction between the tire and the road—provides valuable information to control systems by enhancing the situational awareness of driver-assistance systems and autonomous vehicles. In addition to forces and moments, the estimation of other kinematic parameters—such as the sideslip angle, slip ratio, contact patch length, and the determination of the slip and grip zones within the contact patch, as well as the pneumatic caster distance—along with the estimation of road surface parameters such as the coefficient of road adhesion, is of great importance.

A comprehensive investigation of the smart tire concept was carried out within the APOLLO project [1], which examined the potential of smart tire technologies to improve driving safety. Conducted between 2002 and 2005, the project was coordinated by the Technical Research Centre of Finland (VTT) and involved several universities, automotive manufacturers, and tire manufacturers. Beyond the APOLLO project, other studies reported in the literature can be broadly classified according to the sensing technologies employed. These studies have utilized a wide range of primary sensing technologies, including accelerometers, resistive strain sensors (strain gauges), piezoelectric strain sensors, laser sensors, cameras, and ultrasonic distance sensors. In addition, pressure and temperature sensors have often been incorporated as complementary sensing elements. A comprehensive literature review, including detailed tables summarizing which dynamic and kinematic tire characteristics have been estimated from signals obtained using different measurement techniques, is provided in [2].

In [3], tire pressure variations were used to estimate vehicle mass, with potential applications including driving-range and load-distribution estimation. In [4], accelerometers mounted on the tire were used to estimate vertical force and sideslip angle. In [5], circumferential acceleration signals were used to determine contact-patch length and to identify characteristic changes associated with aquaplaning. More recently, accelerometer-based intelligent tire approaches have also been evaluated under real-road conditions. Wu et al. investigated vertical-load estimation using tire-mounted acceleration measurements and demonstrated the applicability of the approach in road tests under varying operating conditions [6]. In a subsequent study, the same research group developed a dedicated intelligent-tire testing framework extending the evaluation from controlled bench measurements to on-road experiments [7].

Strain-based sensing has also been widely investigated for smart tire applications. Resistive strain gauges have been used on wheel rims, tire structures, and suspension components to estimate quantities such as vertical force, sideslip angle, contact-patch length, wheel speed, and friction-related parameters [8,9,10]. More recently, a strain-based intelligent-tire prototype was investigated under longitudinal-slip conditions, showing that tire-mounted strain measurements can provide useful information across different slip states [11]. However, the limited allowable strain of conventional gauges and long-term bonding durability remain important practical limitations for tire-integrated applications. To overcome these issues, alternative concepts have been proposed in which the tire structure itself acts as a sensing element through deformation-dependent capacitance changes [12,13].

Piezoelectric sensors have also been investigated as an alternative to resistive strain gauges for measuring tire deformation. In [14], a sensor concept was developed to isolate lateral tire deformation from circumferential and radial effects, and the measured response was used to estimate sideslip angle and tire–road friction characteristics. In [15], two piezoelectric sensors were mounted on the tire sidewall at locations determined by finite element simulations, and their voltage outputs were related to wheel force and moment measurements using an artificial neural network. Although the general force and moment trends were captured, the prediction errors remained relatively high compared with the corresponding finite element results.

Optical methods have increasingly been investigated for tire deformation measurement and real-time monitoring. Early studies employed image processing and three-dimensional reconstruction for aircraft tire deformation [16], while other approaches correlated optically measured internal displacements with tire forces [17]. Camera-, thermal-, and laser-based systems were later used to measure local tire deformation, temperature effects, and tread or sidewall displacement [18,19,20].

Stereo-vision methods have been applied to road-profile reconstruction, sideslip-angle estimation, and three-dimensional contact-patch deformation measurement [21,22]. Fiber Bragg grating sensors have also been used to identify contact-patch characteristics from strain-induced wavelength changes [23]. In [24], a rim-mounted high-speed camera was used to track the deformation of a marker grid on the inner tire surface. The measured deformation was used to estimate longitudinal and vertical tire forces with a neural-network-based approach and was compared with corresponding FTire results.

In [25], a stereo digital image correlation (DIC) system was developed to measure the three-dimensional deformation of the tire inner liner. A single wide-view camera combined with two mirrors generated two virtual viewpoints, enabling stereoscopic reconstruction within the limited space inside the wheel. The system was tested on a flat-track rig under different operating conditions; however, the optical arrangement, calibration procedure, and DIC-based post-processing may increase system and computational complexity.

A general assessment of smart tire technologies shows that accelerometer-, strain-, laser-, and ultrasonic-based approaches typically rely on measurements from a limited number of discrete locations. In addition, many of these sensing concepts require sensors, bonded elements, wiring, or other hardware to be attached to or embedded in the tire structure, which may alter the local mechanical behavior of the tire and complicate long-term durability. Such measurements may therefore not fully represent the spatially distributed nature of tire–road interaction. Camera-based approaches, in contrast, can obtain deformation information from the existing tire surface without requiring direct modification of the tire structure itself. This also enables a larger region of the inner liner to be observed simultaneously. However, full three-dimensional reconstruction may require a more elaborate optical setup and more intensive data processing.

Despite the advantages of optical sensing, a gap remains between simple point-based tire sensors and more elaborate full-field optical measurement systems. Conventional embedded or bonded sensors generally provide information from a limited number of locations and may require modifications to the tire structure, whereas advanced camera-based techniques can capture richer deformation fields but often involve complex optical configurations, three-dimensional reconstruction, data-driven force mapping, and substantial computational effort. These requirements limit their practicality for real-time, vehicle-mounted applications. The present study addresses this gap by employing a single high-speed camera and a circular marker grid to extract a limited set of physically interpretable deformation metrics from the tire inner liner without requiring full three-dimensional reconstruction or neural network-based force mapping [24]. Rather than reconstructing the complete deformation field, the proposed approach focuses on simpler geometric quantities that can potentially be related to tire forces, moments, and operating conditions with reduced hardware and computational complexity. The study therefore investigates the static and dynamic behavior of these metrics under variations in vertical load, inflation pressure, maneuver speed, and turn direction, and evaluates their potential for future tire-state and force-estimation applications.

## 2. Materials and Methods

**Figure 1:**
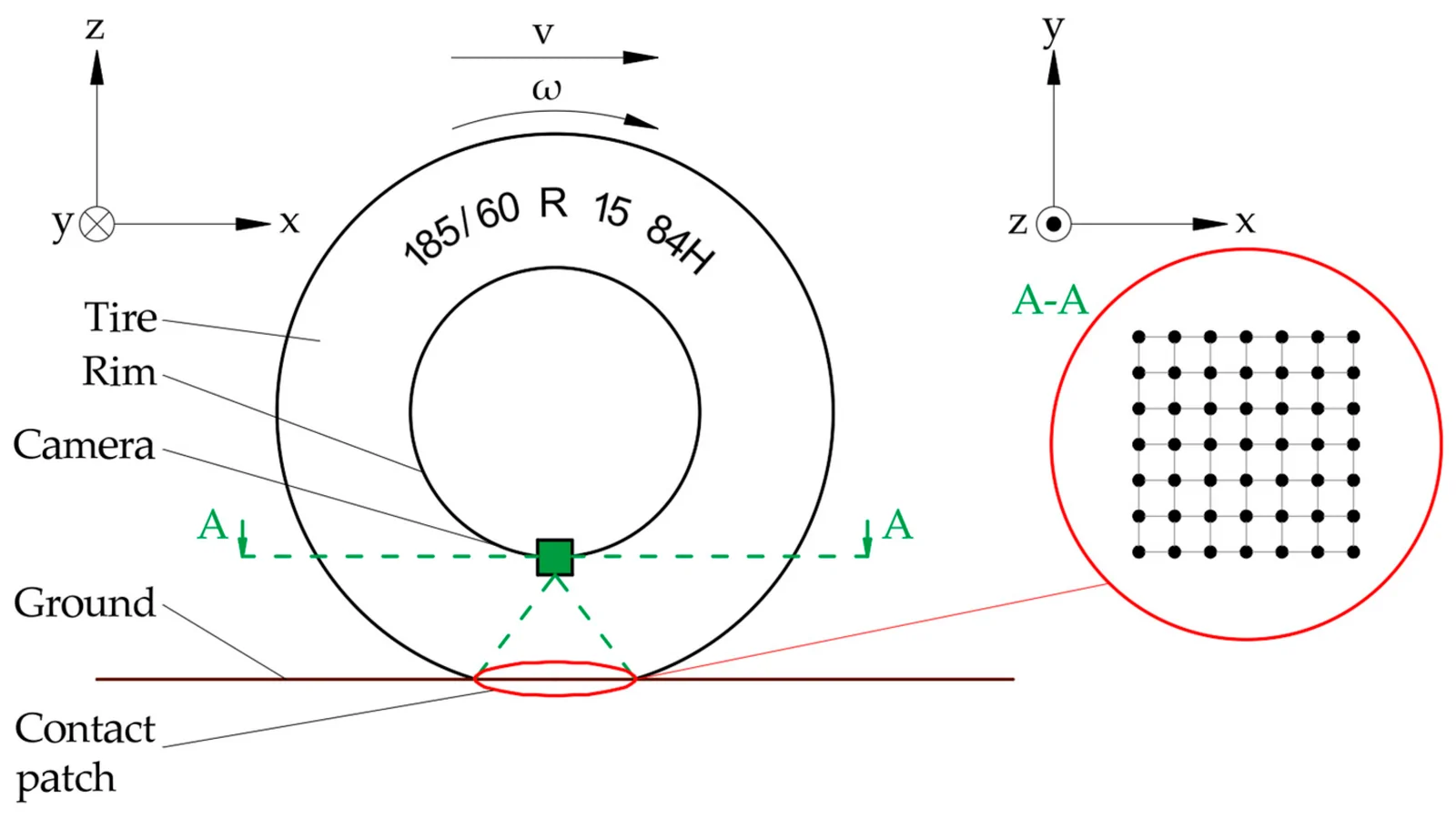
Schematic of the measurement concept.

The proposed method determines the two-dimensional deformation of the tire contact patch by converting the observed area into a 7 × 7 grid of circular markers and tracking the displacements of these markers under the effects of tire forces and moments using a high-speed camera. The grid consisted of 5 mm diameter circles with 15 mm center-to-center spacing in both directions and was applied to the tire inner liner while the tire was in its undeformed state. A schematic representation of the measurement concept is given in Figure 1. Since changes in the apparent diameters of the markers in the two-dimensional images are related to radial deformation of the tire, this information is considered to provide additional sensitivity to out-of-plane deformation.

The remainder of this section is organized into three main parts: the construction of the measurement system, the test systems and experimental procedures, and data acquisition and analysis.

### 2.1. Measurement System

The measurement system consists of three main subsystems: the camera unit, the computer unit, and the wheel unit. The camera unit is installed inside the wheel and comprises the camera, lens, lens mount, illumination components, and associated mechanical supports. The computer unit is located outside the wheel and is responsible for camera control, image acquisition, storage, and processing. The wheel unit consists of a specially manufactured rim designed to accommodate the internal camera assembly and a test tire whose inner liner was prepared as described in Figure 1.

#### 2.1.1. Camera Unit

A key principle adopted in the development of the measurement system was to minimize error sources through the mechanical design and instrumentation itself before relying on software-based compensation. Accordingly, the optical and mechanical components were selected and configured to achieve the highest practical measurement quality at the hardware level, while residual effects were addressed through subsequent image-processing procedures where necessary.

The optical design was based on parameters including field of view, working distance, aperture, depth of field, diffraction effects, modulation transfer characteristics, lens distortion, and the spectral response of the camera sensor [26,27,28,29]. Since the camera operates at a relatively short object distance, the mechanical dimensions of the compound lens and the available installation space inside the wheel were also considered in addition to conventional thin-lens calculations.

**Figure 2:**
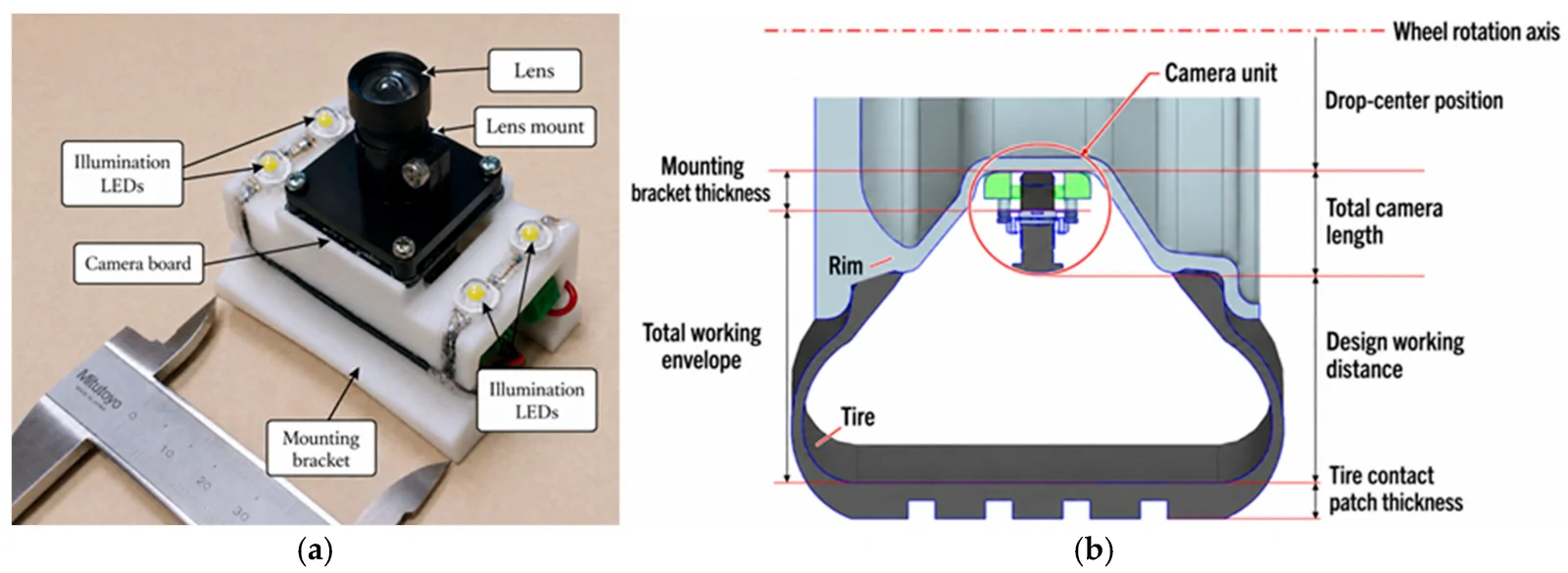
Camera unit and its integration into the test wheel: (**a**) Assembled camera unit. (**b**) Cross-sectional view of the test wheel showing the relevant dimensions and camera-unit position.

Based on these requirements, the DMM 37UX273 camera from The Imaging Source Europe GmbH (Bremen, Germany), equipped with a Sony IMX273LLR-C sensor (Sony Semiconductor Solutions Corporation, Atsugi, Kanagawa, Japan), was selected. The camera provides a full resolution of 1440 × 1080 pixels at up to 238 fps. A global-shutter sensor was preferred to avoid intra-frame geometric distortion caused by rapid changes in the observed deformation field during image acquisition, which could otherwise affect the reconstructed deformation profiles. The camera was combined with a TBN 5.4 10 MP low-distortion S-mount lens to minimize optical distortion. The lens was connected to the camera board through an adjustable lens mount, which provided the required mechanical interface and allowed the focus to be adjusted according to the selected working distance. Illumination was provided by four 1 W blue light-emitting diodes (LEDs) integrated into the camera unit. The camera was mechanically fastened to a dedicated adapter, while the adapter itself was bonded to the rim using Loxeal 31–42 epoxy adhesive (Loxeal S.r.l., Cesano Maderno, MB, Italy). Communication between the camera and the onboard computer was established through a USB 3.1 flat cable. Figure 2a shows the assembled camera unit, while Figure 2b presents a cross-sectional view of the test wheel illustrating the relevant dimensions and the installation position of the camera unit.

Following the mechanical integration of the camera unit, camera calibration was performed to characterize the residual geometric distortion of the assembled optical system and to determine the parameters required for subsequent image correction. The Camera Calibrator app in MATLAB R2025b (Computer Vision Toolbox, version 25.2) was used with checkerboard images acquired at different positions and orientations. A standard camera model with two radial distortion coefficients was adopted, while tangential distortion and image-axis skew were neglected. The calibration procedure provided the intrinsic parameters of the camera, including the focal lengths $f_{x}$ and $f_{y}$, the principal-point coordinates $c_{x}$ and $c_{y}$, and the radial distortion coefficients $k_{1}$ and $k_{2}$. In addition, the extrinsic parameters, represented by the rotation and translation vectors, were estimated independently for each checkerboard pose. All calibration images were successfully used in the parameter estimation. The resulting intrinsic parameters, representative extrinsic-parameter uncertainty metrics, and mean reprojection error are summarized in Table 1. The estimated calibration parameters were subsequently incorporated into the image-processing pipeline to correct lens distortion prior to the deformation analysis.

**Figure 3:**
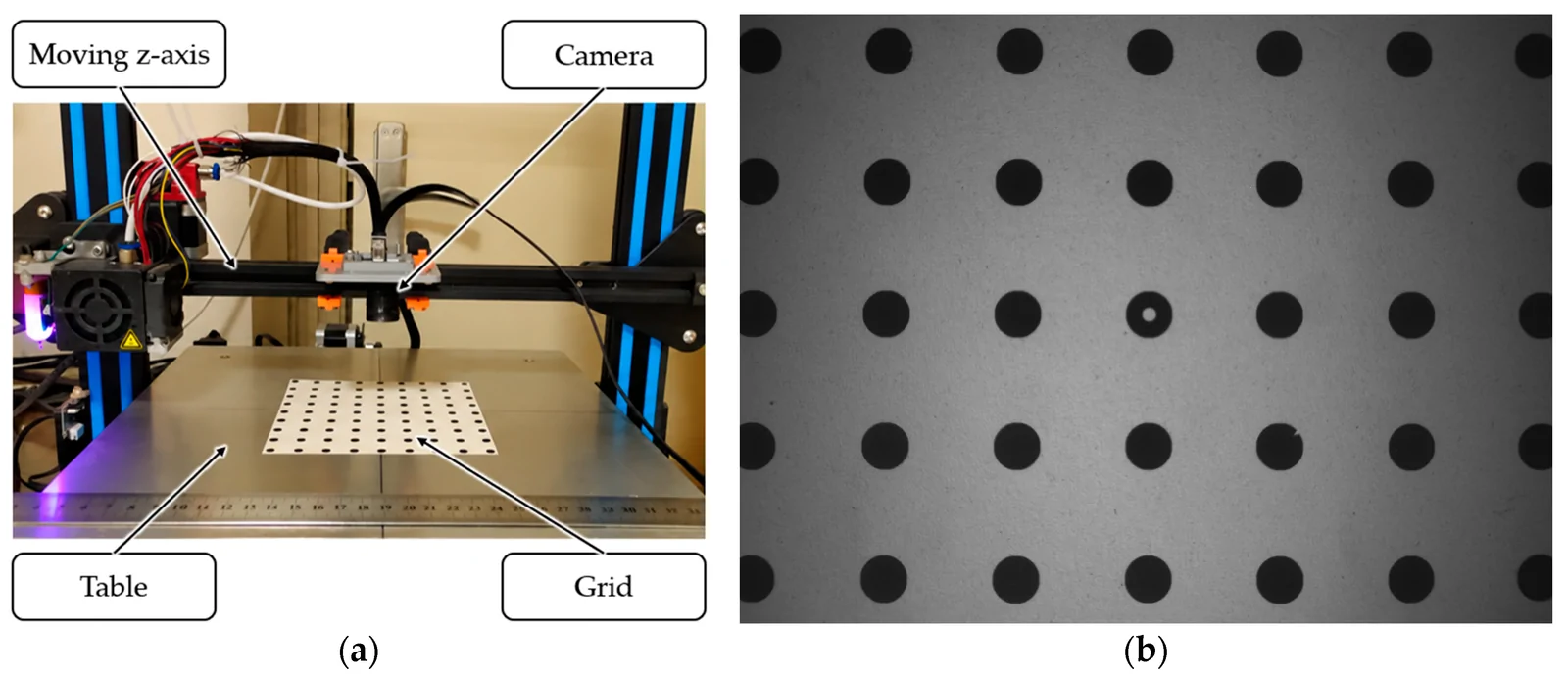
Dimensional validation of the camera-based measurement system: (**a**) Experimental setup used to vary the camera-to-target working distance. (**b**) Representative circular-marker image acquired at a corrected working distance of 71.3 mm.

To assess the dimensional accuracy of the complete image-processing procedure, a planar circular-marker target of known geometry was imaged at different camera-to-target distances using the same camera and optical configuration employed in the measurement system. The target was designed with nominally 5 mm circular markers arranged on a regular grid with a center-to-center spacing of 15 mm in both directions. However, the mean marker diameter measured on the printed validation target using a digital caliper was 5.30 mm, and this measured value was used in the pixel-to-millimeter scale calculation. For each image, lens distortion was first corrected using the previously determined camera calibration parameters. The marker centers and radii were then determined using the Circular Hough Transform (CHT)-based image-processing procedure described in detail in Section 2.3. A geometrically consistent central 3 × 3 marker subset was subsequently identified, and the pixel-to-millimeter scale was determined from the detected marker radii. The dimensional reconstruction was independently evaluated using the two corner-to-corner diagonals of the selected 3 × 3 grid, corresponding to a reference length of 42.426 mm. The validation setup and a representative image acquired at one of the investigated working distances are shown in Figure 3a,b, respectively.

**Figure 4:**
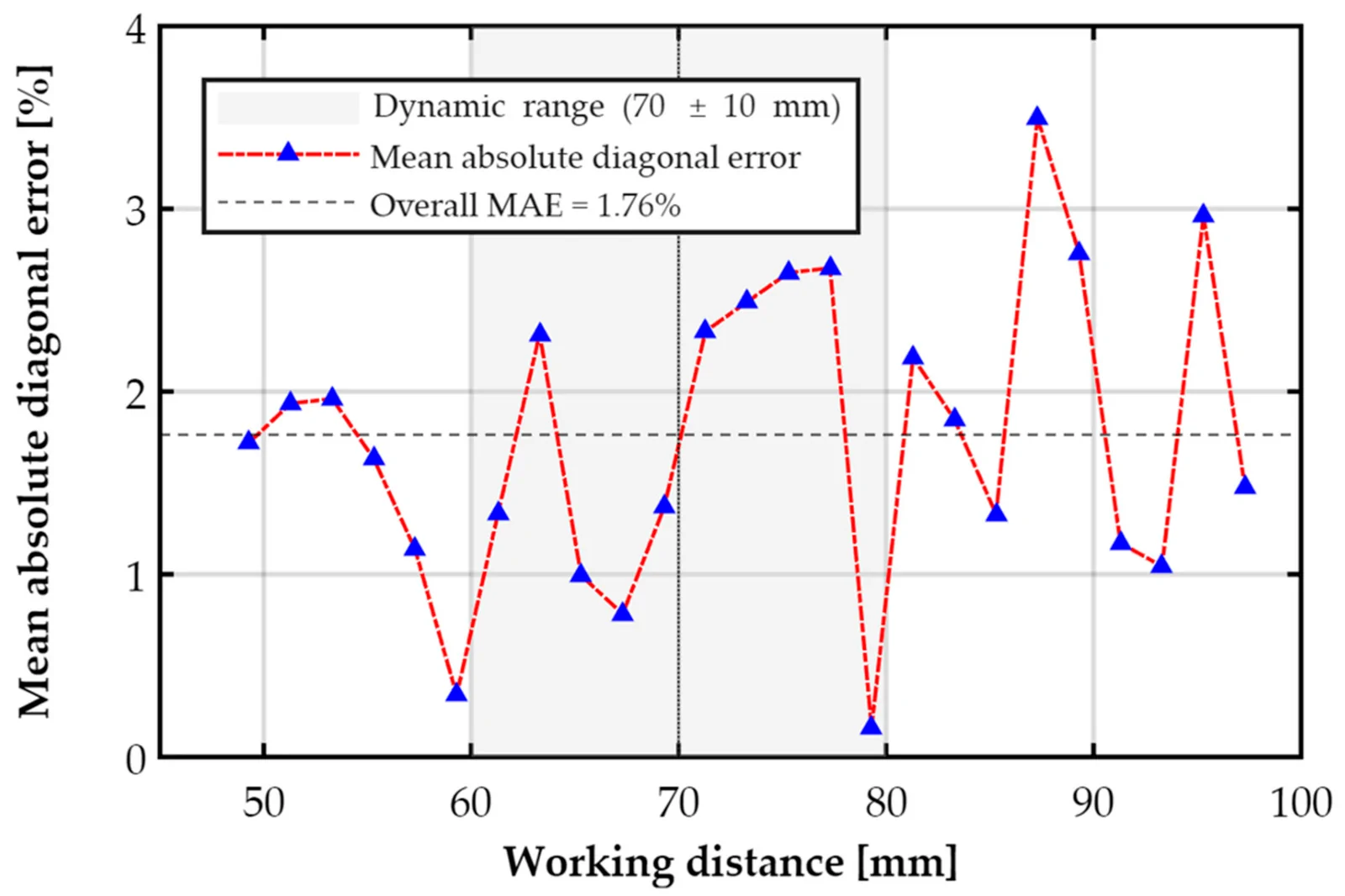
Mean absolute diagonal reconstruction error as a function of working distance. The highlighted region indicates the approximate working-distance range encountered during the dynamic tire measurements (70 ± 10 mm).

A total of 25 images were evaluated over a corrected working-distance range of 49.3–97.3 mm, and all images were successfully processed. This validation interval was intentionally wider than the working-distance range encountered during the dynamic tire measurements, for which the effective camera-to-target distance varied around 70 ± 10 mm. Accordingly, the practically relevant operating interval was approximately 60–80 mm, while the extended validation range was used to assess the robustness of the scale-estimation and dimensional-reconstruction procedure beyond the nominal operating conditions. Over the complete validation range, the radius-based scale varied from 0.0449 to 0.0883 mm/pixel. The mean reconstructed diagonal length was 41.773 ± 0.509 mm, corresponding to a mean dimensional bias of −0.653 mm. The mean absolute error was 0.748 mm (1.76%), while the root-mean-square error was 0.825 mm (1.94%). The maximum absolute error observed over the full validation interval was 1.502 mm (3.54%). The variation in the dimensional error with working distance is presented in Figure 4, where the actual dynamic operating range is highlighted separately. The mean coefficient of variation (CV) of the detected marker radii was 1.28%, and the mean geometric grid-fitting residual was 1.686 pixels, indicating stable marker identification over the investigated distance range.

#### 2.1.2. Wheel Unit

The wheel unit consists of a specially designed test rim and a Goodyear^®^ EfficientGrip 185/60 R15 84 H tire (The Goodyear Tire & Rubber Company, Akron, OH, USA) selected to match the 2006 Toyota Yaris (Toyota Motor Corporation, Toyota City, Aichi, Japan) used in the tests described in Section 2.2.1 and Section 2.2.2. The nominal rim dimensions were based on 5.5J × 15CH ET45, while the rim itself was manufactured from 6061-T6 aluminum alloy. A conventional production rim could not provide the installation space and working distance required by the camera unit; therefore, a custom rim with an enlarged and repositioned drop-center region was developed while retaining, as far as practicable, the bead-seat and sealing geometries required for tire mounting [30,31,32,33].

The relatively deep drop-center geometry was also made possible by the rear drum-brake configuration of the test vehicle, which provided greater available space on the inboard side of the wheel than a disc-brake arrangement would typically allow. Because the modified rim geometry could potentially introduce additional compliance, its structural rigidity was evaluated under representative wheel loads and moments. The calculated displacement of the camera-supporting region remained on the order of hundredths of a millimeter, substantially below the tire deformation levels of interest. The minimum wall thickness in the rim-well region was 5.5 mm, and the final rim was machined from solid stock because its geometry was not suitable for conventional low-pressure casting.

**Figure 5:**
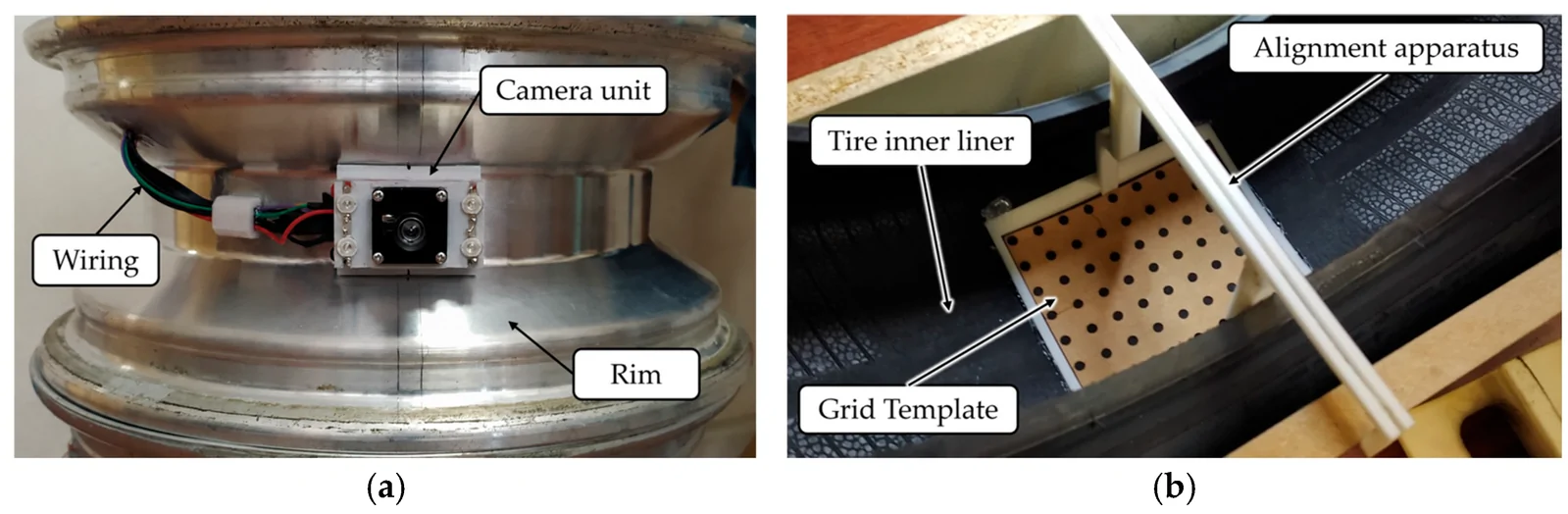
Wheel unit: (**a**) Test rim with the camera unit installed. (**b**) Preparation of the deformation grid on the test tire.

For optical tracking, a grid of circular markers was applied to the tire inner liner within the camera field of view. The marker pattern deforms together with the tire, enabling the deformation of the observed region to be quantified from the recorded images. Figure 5a shows the test rim with the camera unit installed, whereas Figure 5b illustrates the preparation of the deformation grid on the inner liner of the test tire using the alignment apparatus and grid template.

#### 2.1.3. Computer Unit

The computer unit is responsible for controlling the camera and illumination system, providing interfaces for auxiliary sensors, and housing the onboard power supply and associated power-conditioning electronics. The main processing platform is an NVIDIA Jetson Nano (NVIDIA Corporation, Santa Clara, CA, USA), accompanied by two Arduino Nano boards (Arduino S.r.l., Monza, Italy). One Arduino is reserved for interfacing with auxiliary sensors, such as the optical incremental encoder and other sensors that may be activated in future configurations, whereas the second controls a custom-built infrared remote-control circuit used for switching the system power and illumination on and off. The unit is powered by a four-cell LiPo battery (Power-Xtra PX2200HP4S; Birikim Pilleri Batarya Sanayi Tic. Ltd. Şti., Istanbul, Türkiye). Since the NVIDIA Jetson Nano requires a regulated 5 V supply; an XL4015 DC–DC step-down voltage regulator (XLSEMI, Shanghai, China) is used to provide the required supply voltage from the battery. The illumination system is driven by a 700 mA constant-current LED driver to provide a stable current to the LED array. With the illumination active and the camera operating at a moderate acquisition rate, the battery provides approximately 2 h of operation. The electronics and battery are housed in a cylindrical enclosure manufactured from polylactic acid (PLA) filament using fused-filament 3D printing and mounted externally on the wheel rim.

**Figure 6:**
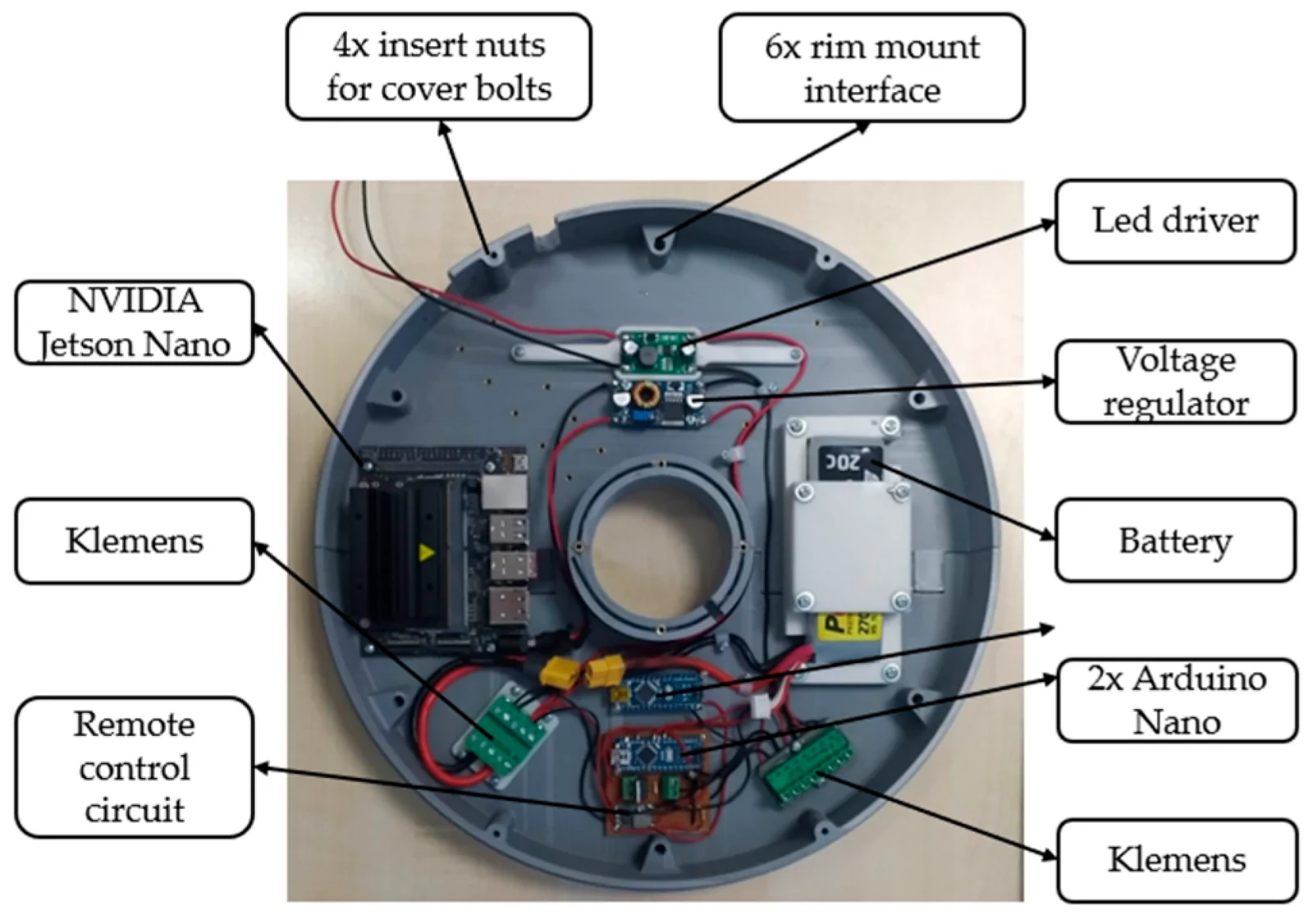
Assembled components of the computer unit inside the enclosure.

A cable bundle connects the computer unit to the camera and illumination components located inside the wheel through a single opening machined in the rim. After the cables were routed through this opening, the remaining clearance around the cable bundle was filled with Loxeal 31–42 epoxy adhesive to maintain the pressure tightness of the wheel. Additional sealing was also applied inside some cable jackets, since air leakage was observed through the internal gaps between the conductors and the outer insulation. The manufactured and assembled computer unit is shown in Figure 6.

### 2.2. Test Systems and Procedures

The experimental program was conducted in two stages. First, static tests were performed under controlled loading conditions to evaluate the response of the optical measurement system to known changes in vertical load and inflation pressure. Subsequently, dynamic tests were carried out on an instrumented test vehicle to investigate whether the proposed concept could acquire consistent and usable deformation data under realistic rolling conditions. This two-stage approach therefore addressed both the controlled characterization of the measurement principle and its practical applicability under vehicle operation.

#### 2.2.1. Static Test Setup and Procedure

**Figure 7:**
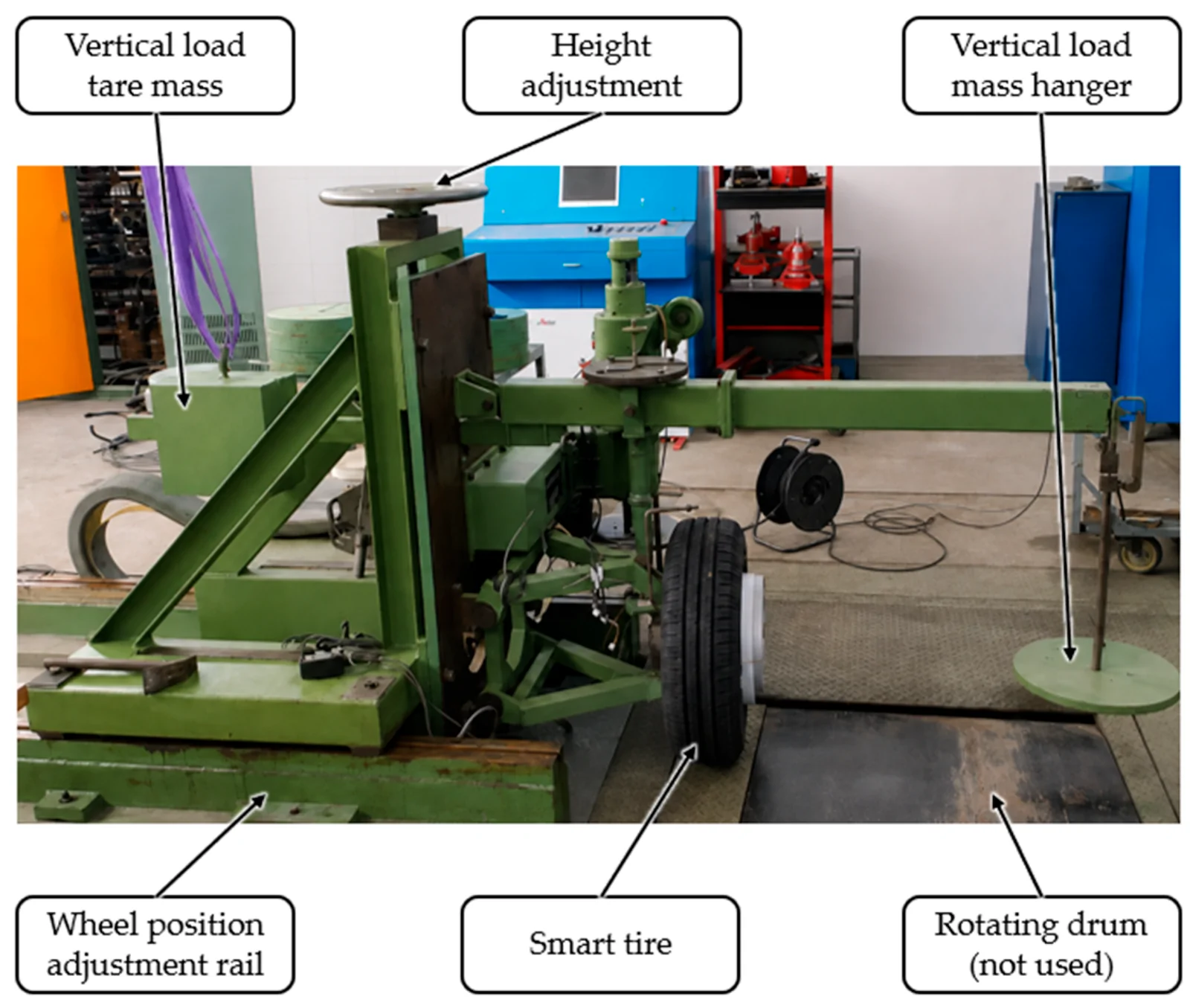
SCHENK Drum Type Tire Testing System used in static tests as a loading platform.

For the static tests, a drum-type tire test system (SCHENK; Carl Schenck AG, Darmstadt, Germany)was used as a controlled loading platform, while the tire itself was pressed against a flat surface rather than the drum, as shown in Figure 7. Tests were conducted at inflation pressures of 28, 32, and 36 psi, with the camber angle fixed at 0°. At each pressure level, the vertical load was varied from 0 to 4000 N in increments of 250 N, and images of the marker grid were acquired at each loading condition.

The static tests were primarily intended to characterize the deformation response measured by the optical system under well-defined loading conditions before proceeding to vehicle-based dynamic measurements. Although the test setup was originally intended to allow a wider range of operating conditions, the present study was limited to zero camber during the static test campaign.

To extend the static evaluation to the vehicle-installed configuration, an additional multi-point vehicle-level validation campaign was performed using the instrumented right-rear wheel of the test vehicle. The tire inflation pressure was maintained at 32 psi. The vertical wheel load was measured using an MWT Load Meter portable wheel-load scale (MWT Meß- und Wiegetechnik GmbH & Co. KG, Wennigsen, Germany) positioned beneath the instrumented wheel, while a static image of the deformation grid was acquired simultaneously for each loading condition. Wheel loads above the unloaded static-equilibrium condition were generated by placing calibration masses of different sizes at varying positions on the right-rear seat and in the luggage compartment. For wheel loads below the unloaded static-equilibrium condition, the rear axle was progressively supported using a hydraulic jack, thereby reducing the load carried by the instrumented wheel.

The resulting wheel-load levels were not uniformly spaced because the added calibration mass does not translate directly into an equal increment in the measured wheel load. The load transferred to the instrumented wheel depends on the position of the added mass and on the resulting changes in vehicle body attitude, suspension deflection, and overall load distribution. Therefore, the wheel-load value measured by the portable scale, rather than the nominal added mass, was used as the reference vertical load for each condition. The corresponding optical vertical-load estimate was subsequently obtained from the diagonal-distance metric using the 32 psi static calibration established on the controlled tire-test system.

Repeatability was evaluated at three prescribed static wheel-load levels. For each load level, the vehicle was first brought to the target condition through an appropriate combination of calibration-mass placement and partial support of the rear axle using the hydraulic jack. Once the target condition had been established, one of the calibration masses was temporarily removed to disturb the loading state and, after a fixed settling interval, replaced in its original position. A new image of the deformation grid was then acquired after the system had settled. This unloading-reloading procedure was repeated ten times for each of the three load levels, providing independent realizations of the same nominal loading condition for the repeatability assessment.

#### 2.2.2. Dynamic Test Setup and Procedure

For the dynamic tests, the test vehicle integrated with the measurement system was used. A VBOX VB20SL (RACELOGIC Ltd., Buckingham, UK) global positioning system (GPS) was installed on the test vehicle to obtain vehicle-motion data. In the dual-antenna configuration, the VBOX determines vehicle heading from the relative positions of the two antennas and provides yaw-rate and slip-angle information. The system can additionally translate the measured slip angle from the reference-antenna location to predefined positions on the vehicle using the corresponding longitudinal and lateral offsets [34]. In the present study, the right-rear local slip-angle channel, α_RR_, was provided directly by the VBOX processing unit rather than being calculated separately from the recorded signals. One of the GPS antennas was positioned approximately above the right-rear wheel using a dedicated suction-cup-mounted bracket. The static toe angle of the rear wheels was measured to be approximately 0°. Since the vehicle is equipped with a twist-beam rear suspension without rear-wheel steering, and the associated elastokinematic steer was assumed to be small under the investigated operating conditions, the VBOX-derived right-rear local slip angle was taken as an approximation of the right-rear tire slip angle. Dynamic toe variation was not measured independently; therefore, this equivalence should be interpreted as an engineering approximation rather than an exact wheel-plane measurement.

The dynamic tests consisted primarily of constant-radius cornering (CRC) maneuvers performed on an approximately 40 m diameter circular path marked in an open parking area in İstanbul Technical University, Ayazağa Campus. The vehicle was driven at nominal constant speeds from 10 to 40 km/h in 5 km/h increments, corresponding to approximately 0.4 to 6.2 m/s^2^ of lateral acceleration a_y_, for both left- and right-hand cornering and at each of the three tire inflation pressures used also in static tests. Representative rim-mounted camera recordings from the dynamic tests are provided as Supplementary Videos S1–S42. In addition, straight-line tests were performed at 10, 20, and 30 km/h. Considering all cornering and straight-line conditions, a total of 51 test cases were evaluated.

Prior to data acquisition, the tires were warmed so that the tread surface temperature remained approximately within 45–50 °C during the tests, thereby limiting the associated inflation-pressure variation to approximately 1 psi. Since the vehicle speed and path radius were approximately constant during each CRC maneuver, the tests were treated as quasi-steady-state, and the tire forces and sideslip angle at the measured wheel were assumed to remain approximately constant over the data-acquisition interval.

**Figure 8:**
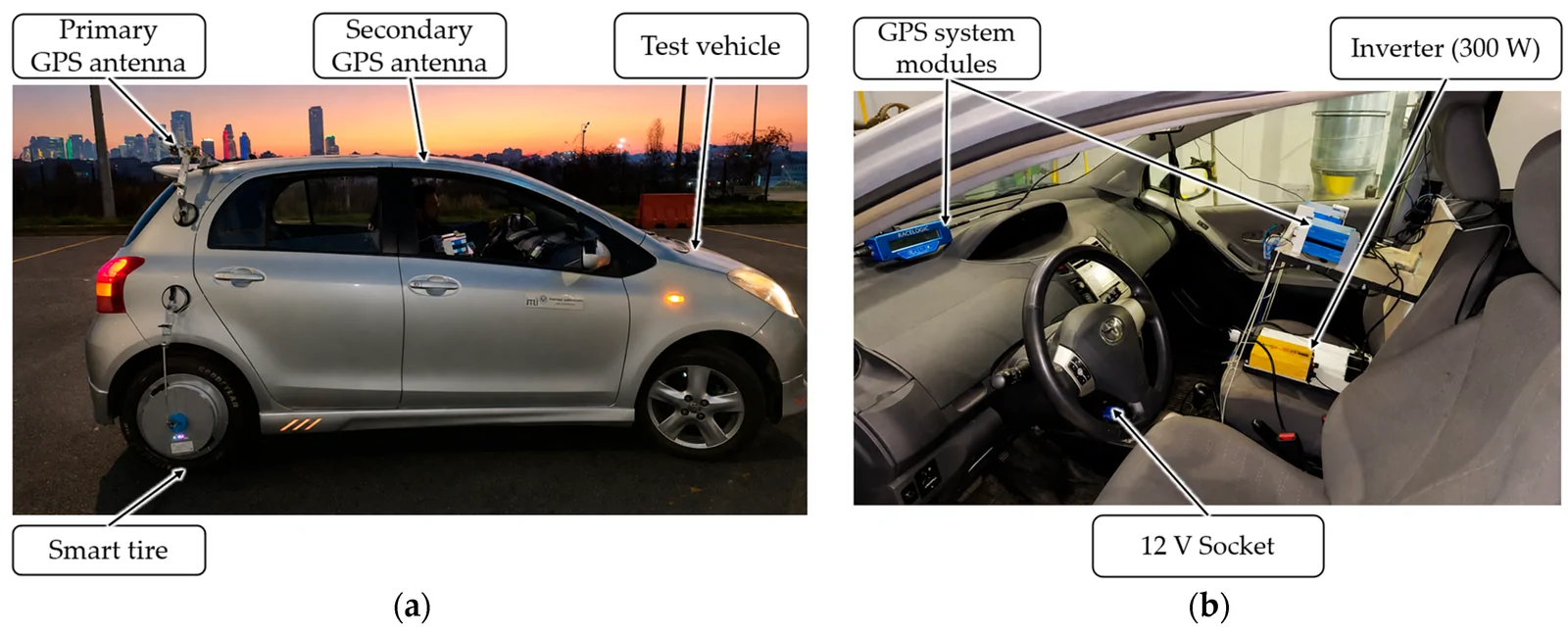
Instrumentation of the dynamic test vehicle: (**a**) External configuration showing the smart tire and GPS antennas. (**b**) Internal configuration showing the GPS system modules, inverter, and 12 V accessory socket.

For each test case, high-speed images were recorded for approximately 3 s over a selected section of the test path with relatively low surface roughness and minimal unevenness. This section was chosen to reduce cycle-to-cycle variations in the measured deformation signals and to maintain conditions as close as possible to a pure cornering state during the constant-radius cornering maneuver. The maximum practical camera acquisition rate was used, varying between approximately 130 and 160 fps. Image acquisition was controlled remotely from a laptop computer connected to the NVIDIA Jetson Nano through a shared Wi-Fi network. The acquisition software, implemented in C++14 and previously installed on the Jetson Nano, was executed remotely via an SSH connection from the laptop. In parallel, GPS data were acquired independently on the same laptop under the Windows operating system using the VBOX Test Suite software (version 2.3.12.6645). Figure 8a,b shows the external and internal configurations of the fully instrumented test vehicle, respectively. In addition to the components visible in the figure, a laptop and a dedicated laptop stand were installed on the rear seat for system control and data monitoring. All onboard equipment, including the GPS modules, inverter, computer, and associated cabling, was securely fixed to prevent unwanted movement and to ensure safe operation during the test maneuvers.

**Figure 9:**
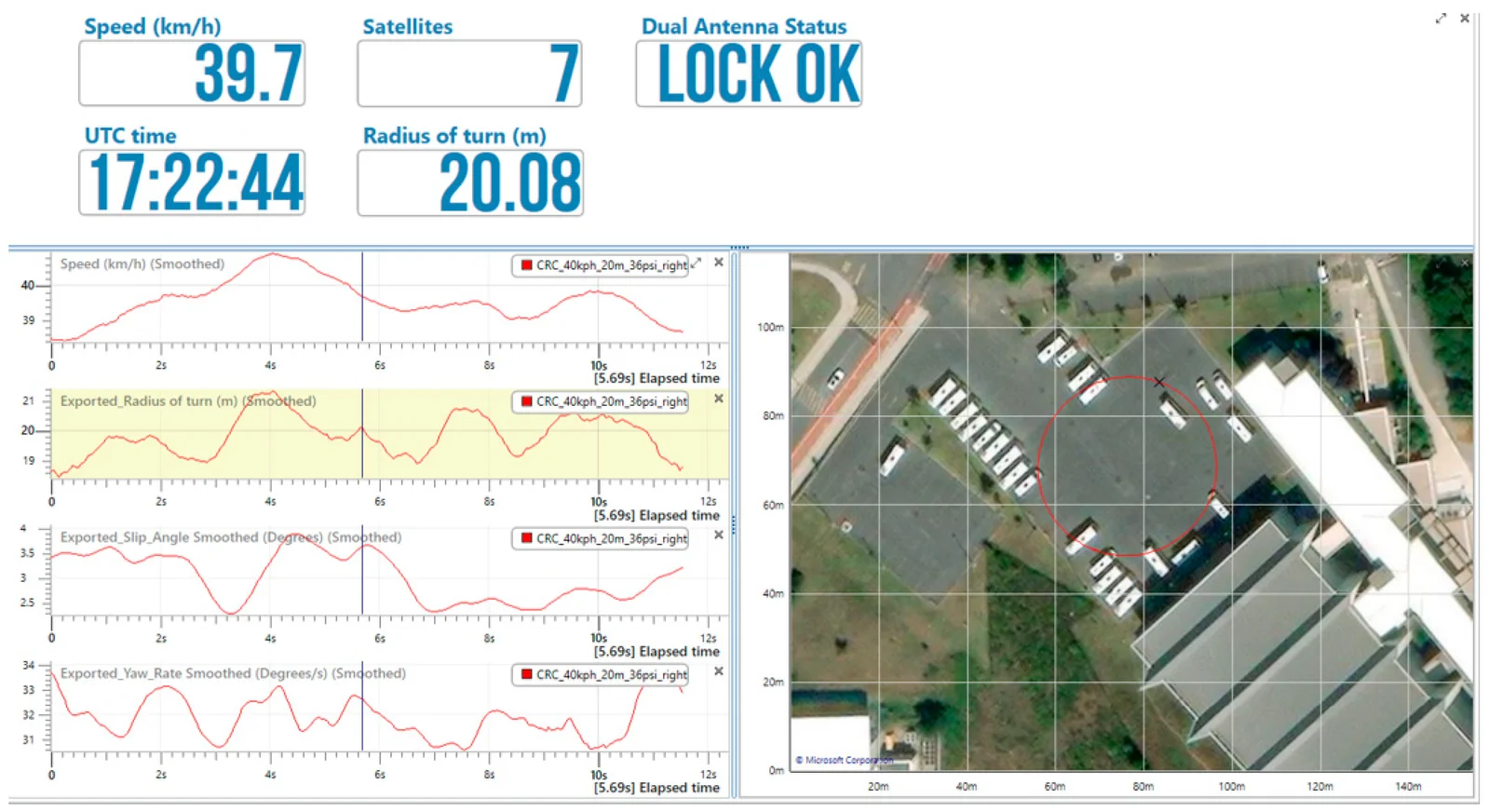
VBOX Test Suite interface used during the dynamic tests, showing the measured quantities, dashboard information, and real-time vehicle trajectory. The red circle indicates the prescribed circular test trajectory.

For each condition, GPS measurements were collected over three complete laps. The software interface provided real-time visualization of the measured quantities together with a dashboard display and the vehicle trajectory overlaid on a map, as illustrated in Figure 9. Because the GPS measurements were susceptible to environmental disturbances, particularly signal degradation caused by surrounding structures, the recorded data were subsequently processed and filtered, and representative mean values were determined for each test case.

### 2.3. Data Acquisition and Analysis

The raw outputs of the smart-tire measurement system were recorded as sequential BMP image files. The first step in the analysis was the digitization of the marker grid. For this purpose, the circular markers were detected using the Circlular Hough Transform (CHT) [35], implemented using the imfindcircles function in MATLAB^®^ R2025b (MathWorks, Natick, MA, USA). Static contact-length measurements were compared with the physical span of the marker pattern before the dynamic analysis. This comparison showed that the full marker field could not be assumed to remain within the contact region, whereas the central 3 × 3 subset provided a consistent observation region within the estimated contact length. The analysis was therefore restricted to these nine markers.

**Figure 10:**
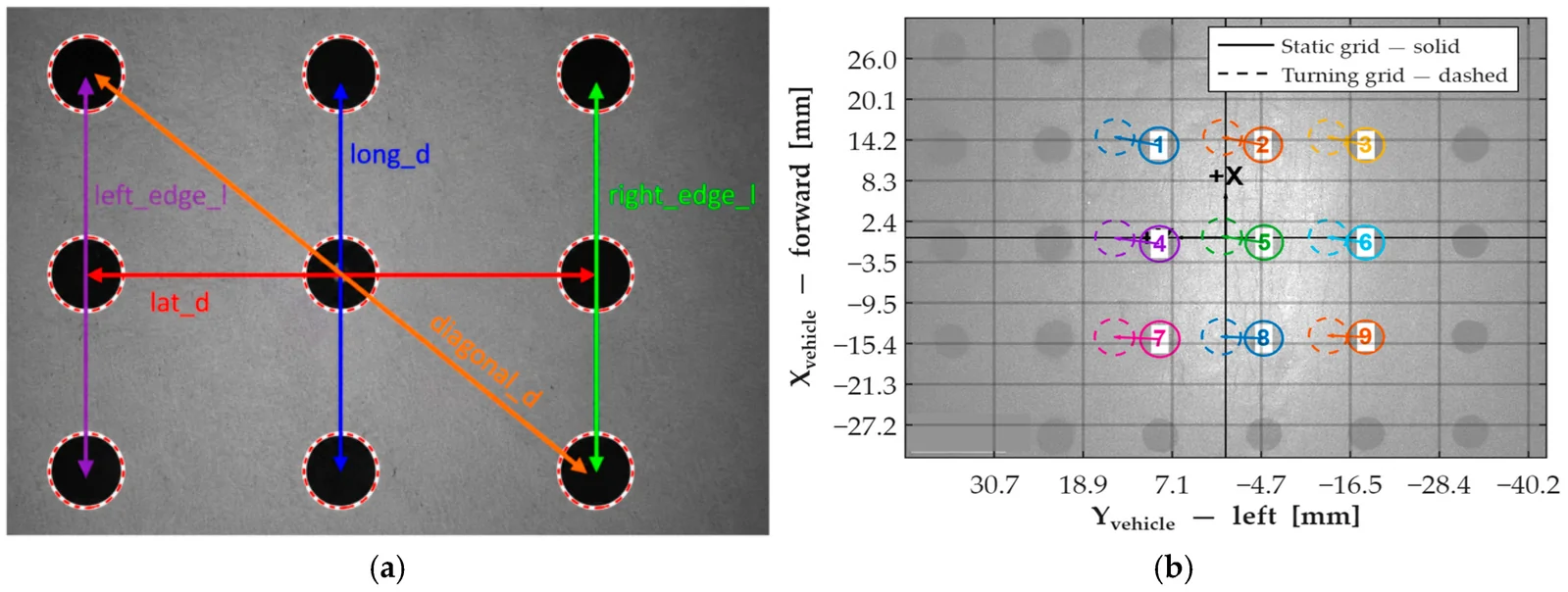
Contact-frame-based analysis at 32 psi tire pressure: (**a**) Geometric metrics derived from the 3 × 3 marker grid. (**b**) Static and 20 km/h left-turn grid configurations under the same vehicle instrumentation and loading conditions.

In the first approach, only the image frames corresponding to the deformation grid–road contact event were considered. Several geometric metrics were calculated from the instantaneous configuration of the 3 × 3 marker grid, including the radius of the center marker (r_center), the lateral and longitudinal centerline distances (lat_d and long_d), the left- and right-edge lengths (left_edge_l and right_edge_l), the corresponding average marker radii along these edges (left_edge_r_avg and right_edge_r_avg), the diagonal distance (diagonal_d), and the average marker radius over the grid (radius_avg). Examples of the distance-based metrics are illustrated in Figure 10a. These metrics were selected to describe both in-plane deformation of the marker pattern and changes in the apparent marker size associated with radial tire deformation.

Figure 10b presents a representative comparison between the static grid configuration and the corresponding grid obtained during a 20 km/h left-turn maneuver at 32 psi inflation pressure, using the same vehicle instrumentation and loading configuration adopted in the dynamic tests. Under left cornering, the tire undergoes lateral deformation toward the right relative to the wheel, which appears in the camera-fixed coordinate system as a displacement of the observed deformation grid toward the left. This example qualitatively illustrates the sensitivity of the proposed image-based approach to lateral tire deformation under cornering.

Although the present measurements were performed at zero camber, the separate left- and right-side metrics may also provide useful information on asymmetric tire deformation. In particular, differences between left_edge_l and right_edge_l, together with differences between left_edge_r_avg and right_edge_r_avg, may potentially be related to camber-induced deformation and could be investigated in future work. The remaining distance- and radius-based metrics are primarily sensitive to changes in tire deformation and camera-to-inner-liner distance and therefore provide multiple complementary descriptors of the contact-region geometry.

In the second approach, the complete time histories of the detected marker coordinates and radii were processed. Since the raw image-processing outputs are expressed in pixels, a local pixel-to-length scale was first determined for each row of the marker grid. The nominal marker diameter, *D*, was known from the applied pattern, while the apparent diameter of each detected marker was obtained from its CHT radius. For row *j*, the instantaneous scale factor was therefore calculated from the mean apparent marker diameter as

(1) $$s_{j}\left(t\right)=\frac{D}{d_{j,px(t)}}\;[mm/pixel\;],$$

(2) $${\bar{d}}_{j,px}\left(t\right)=\frac{1}{N_{j}}\sum_{i=1}^{N_{j}}2r_{ij}(t),$$

where *r_ij_* (t) is the detected radius, in pixels, of marker *i* in row *j*, and *N_j_* is the number of markers in that row. A row-wise rather than a single-marker scale was used to reduce the sensitivity of the conversion to local detection errors and to asymmetric changes in apparent marker size. In particular, averaging the markers across each row reduces the influence of lateral differences in camera-to-surface distance that may arise from tire orientation or camber.

The pixel displacements of each marker relative to the selected reference condition were then converted into absolute displacements using the corresponding row-wise scale:

(3) $$∆X_{ij}\left(t\right)=s_{j}\left(t\right)(x_{ij}(t)-x_{ij,ref}),$$

(4) $$∆Y_{ij}\left(t\right)=s_{j}\left(t\right)(y_{ij}(t)-y_{ij,ref}),$$

where *X* and *Y* denote the longitudinal and lateral directions, respectively. This local scaling approach was preferred over the use of a single image-wide conversion factor because the apparent size of the marker pattern changes with the local camera-to-inner-liner distance. The detected marker radii were also retained as independent signals, since their variation provides information associated with radial deformation and the corresponding change in working distance.

Representative signals from a selected tire pressure of 28 psi, 20 km/h left-turn CRC test case are presented separately for the longitudinal displacement, lateral displacement, and apparent marker radius. For each quantity, the image-processing output expressed in pixels is shown together with its corresponding representation in millimeters after application of the local scaling procedure described above. These comparisons illustrate how the proposed pixel-to-length conversion transforms the measured image-domain quantities into physically interpretable deformation measures while preserving their characteristic temporal behavior.

**Figure 11:**
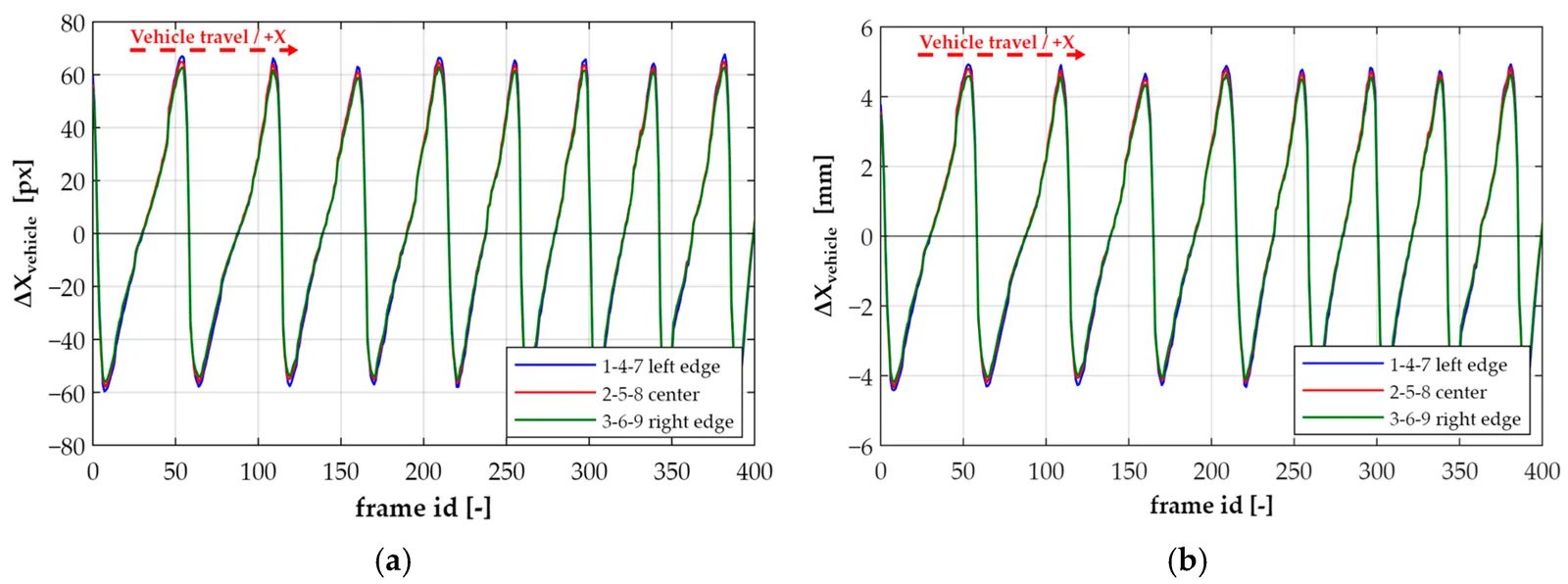
Representative longitudinal displacement signals for 28 psi inflation pressure, 20 km/h left-turn CRC test case: (**a**) Displacement obtained from the image-processing output in pixels. (**b**) Corresponding displacement in millimeters after application of the local pixel-to -length conversion. Left, center, and right denote the three lateral columns of the 3 × 3 analysis grid.

The longitudinal displacement signals are shown in Figure 11. The displacement obtained directly from the detected marker coordinates in pixels is presented in Figure 11a, whereas the corresponding displacement after conversion to millimeters is shown in Figure 11b. The left, center, and right signals correspond to the three lateral columns of the 3 × 3 analysis grid and therefore represent the longitudinal motion of three different lateral regions of the tire inner liner as the grid passes through the contact region. The positive X-direction was defined as the vehicle travel direction, as indicated in the figures. Increasing frame number therefore represents successive instants during forward vehicle motion and the corresponding progression of the observed tire region through the contact zone.

**Figure 12:**
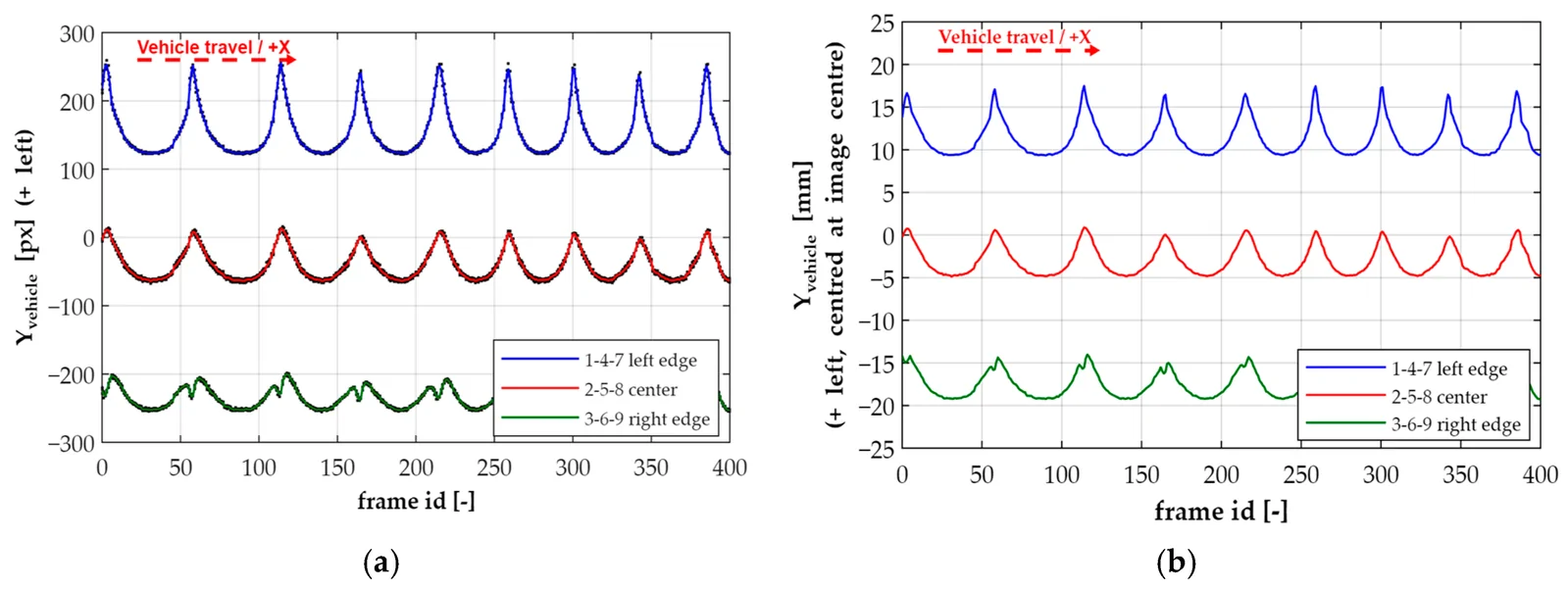
Representative lateral displacement signals for 28 psi inflation pressure, 20 km/h left-turn CRC test case: (**a**) Displacement obtained from the image-processing output in pixels. (**b**) Corresponding displacement in millimeters after application of the local pixel-to-length conversion. Left, center, and right denote the three lateral columns of the 3 × 3 analysis grid.

The corresponding lateral displacement signals are presented in Figure 12. The pixel-domain response is shown in Figure 12a, while its millimeter-domain representation is given in Figure 12b. Retaining separate left, center, and right signals makes it possible to preserve differences in deformation across the tire width rather than reducing the measured response to a single representative trajectory. A comparable distinction between left, central, and right regions was adopted by Zappa et al. [25], whose stereo-DIC measurements also revealed location-dependent lateral deformation of the tire inner liner. The characteristic cyclic behavior observed in the present displacement signals is qualitatively consistent with the inner-liner deformation trajectories reported in that study.

**Figure 13:**
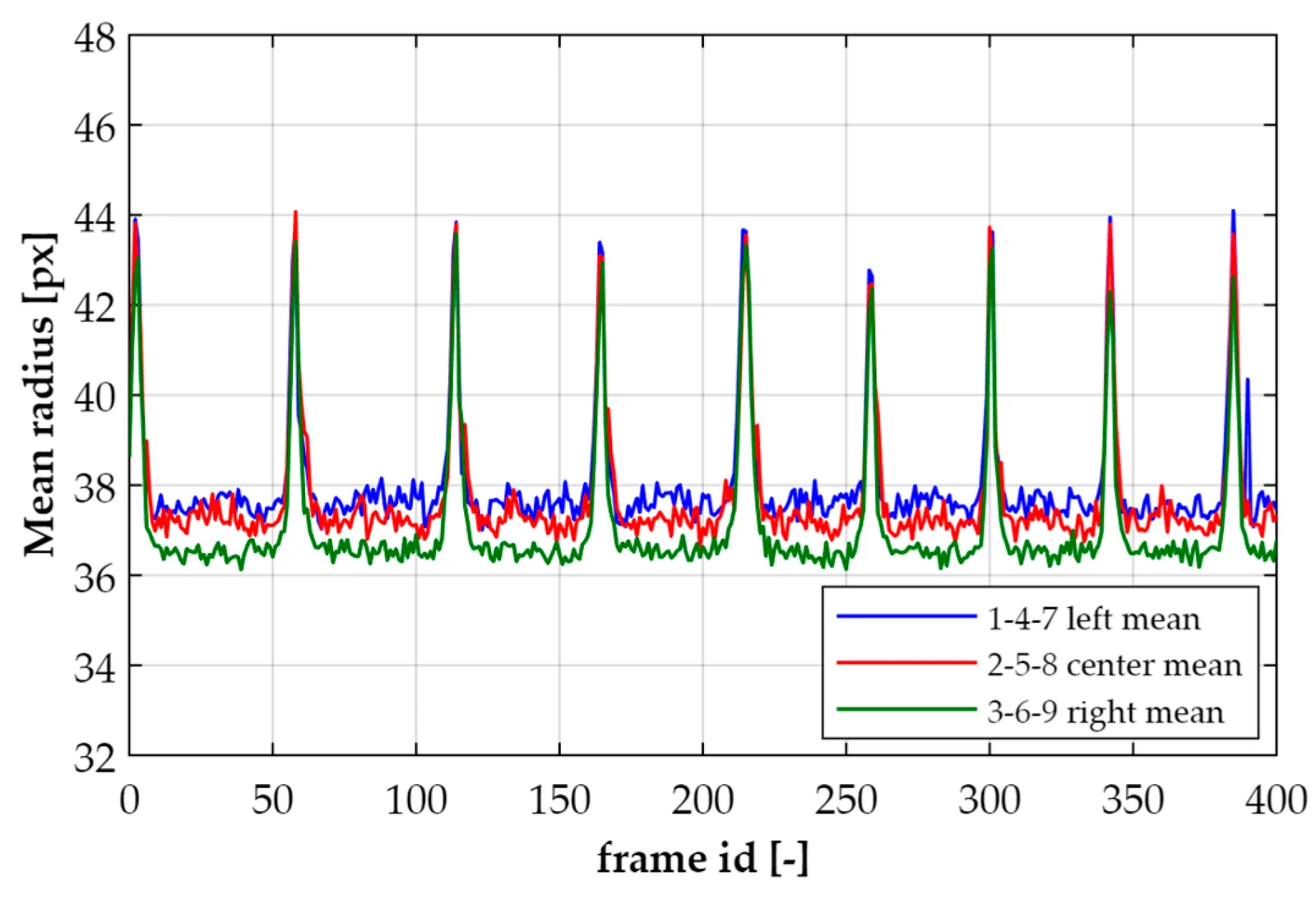
Representative apparent marker-radius signals, expressed in pixels, for 28 psi inflation pressure, 20 km/h left-turn CRC test case. Left, center, and right denote the three lateral columns of the 3 × 3 analysis grid.

The apparent marker-radius signals are presented in Figure 13 and are retained in pixels. The physical marker size is fixed by the applied pattern and therefore does not itself represent a deforming length scale. Instead, variations in the detected radius reflect changes in the apparent marker size in the camera image. Since the apparent size depends on the local camera-to-inner-liner distance, the radius signal provides an additional indicator of radial tire deformation and changes in working distance.

Since the measured deformation responses are periodic with wheel rotation, the subsequent analysis was performed on a cycle basis. The center frame of each tire–road contact event was used as the reference point for the corresponding cycle. Complete cycles were bounded by the temporal midpoints between consecutive contact frames, and only contact events having valid preceding and following boundaries were included in the analysis. Following frame-level signal repair, each candidate cycle was screened for abrupt and non-physical point-to-point discontinuities in the three lateral-column signals obtained from markers 1–4–7, 2–5–8, and 3–6–9. Such discontinuities can arise from occasional marker-grid identification errors; therefore, cycles exceeding the predefined signal-continuity thresholds in any of the three column signals were rejected. The grid-geometry validity score was retained as a diagnostic quantity but was not used as an independent cycle-rejection criterion. The accepted cycles were mapped onto a common normalized coordinate, τ, ranging from −1 to 1. The representative longitudinal curve was obtained by taking the arithmetic mean of the accepted normalized curves at each value of τ. For the lateral analysis, each cycle was first referenced to its off-contact response, and the representative curve was obtained using the pointwise median to reduce sensitivity to residual outlying profiles. The longitudinal amplitude was subsequently calculated from the representative center-line curve as one half of its peak-to-peak excursion, while the lateral metric was defined as the maximum deformation of the representative center-line curve. Thus, these scalar quantities were extracted from the representative curves rather than calculated as the arithmetic means of the corresponding single-cycle amplitudes or peaks. This representation reduces cycle-to-cycle variability and measurement noise while preserving the repeatable deformation pattern associated with one wheel revolution. A conceptually similar strategy was employed in [25], where measurements from more than 100-wheel revolutions were consolidated into a representative single-cycle response to improve the effective angular resolution of the tire inner-liner measurements.

**Figure 14:**
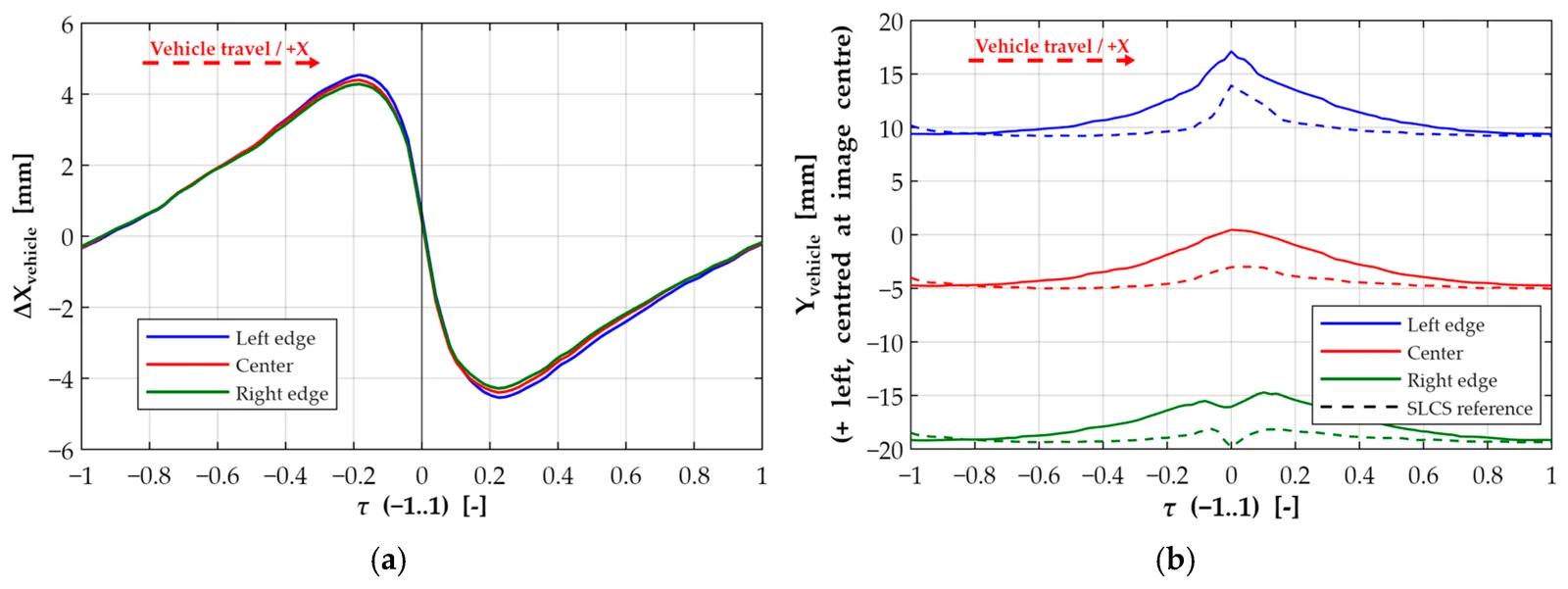
Representative average-cycle responses for 28 psi inflation pressure, 20 km/h left-turn CRC condition: (**a**) Longitudinal deformation. (**b**) Lateral deformation together with the same-speed and same-pressure SLCS reference. Left, center, and right denote the three lateral columns of the 3 × 3 analysis grid, and τ represents one complete wheel-rotation cycle.

Representative average-cycle responses for the longitudinal and lateral deformation are presented in Figure 14a,b, respectively, for 28 psi inflation pressure, 20 km/h left-turn CRC condition. In the longitudinal direction, the left, center, and right signals represent the three lateral columns of the 3 × 3 analysis grid and therefore describe the longitudinal deformation histories of three distinct lateral regions of the tire inner liner. The resulting waveform provides a compact representation of the circumferential evolution of longitudinal deformation during one wheel revolution.

For the lateral deformation, the corresponding average CRC cycle is additionally compared with an average cycle obtained from a straight-line constant-speed (SLCS) test performed at the same inflation pressure and vehicle speed, as shown in Figure 14b. The SLCS response provides a rolling reference containing deformation already present at the same rotational speed. The center signal oscillates around an offset of approximately −5 mm rather than around zero. This offset is attributed primarily to geometric asymmetry introduced by the modified rim drop-center geometry required by the manufacturing and packaging constraints of the measurement system, together with residual centering error introduced during application of the marker pattern to the tire inner liner. Since these effects are largely common to both the CRC and SLCS measurements, the use of the corresponding SLCS response provides a more appropriate baseline than an assumed zero-deformation reference.

The SLCS response also reveals the characteristic lateral deformation produced as the observed grid enters and passes through the contact region. In particular, the separation between the left and right signals increases around the contact event, indicating a lateral stretching of the observed inner-liner region associated with tire flattening under vertical loading. During the CRC maneuver, the deformation associated with the lateral tire force is superposed on this rolling-contact deformation pattern, producing an additional asymmetric lateral displacement of the grid. The resulting CRC response may therefore be interpreted as the combined effect of the baseline deformation associated with rolling contact and the additional lateral deformation generated during cornering, rather than as a completely independent deformation mode.

## 3. Results

### 3.1. Static Tests

**Figure 15:**
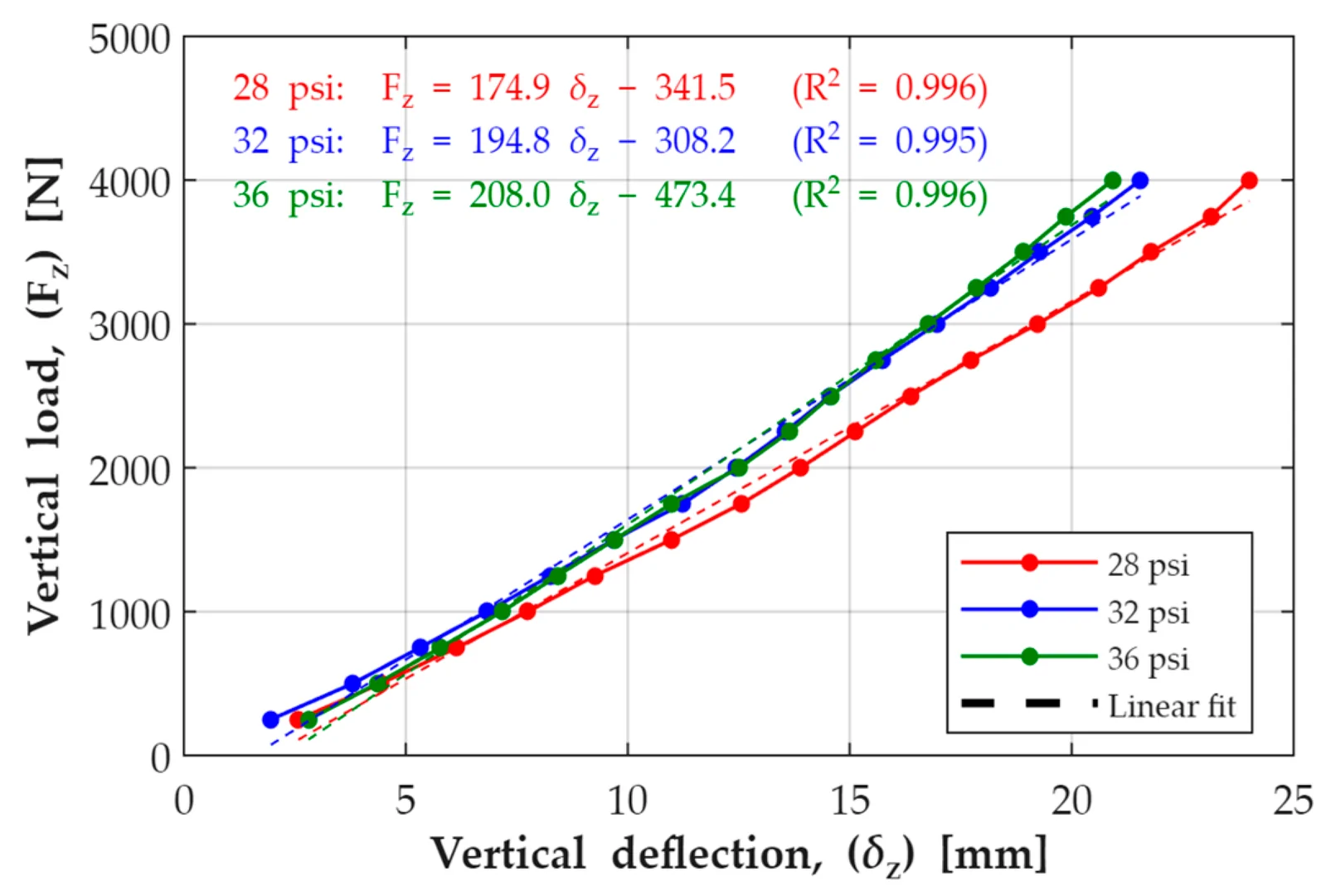
Static vertical load–deflection characteristics of the test tire at inflation pressures of 28, 32, and 36 psi under 0° camber.

Static tests were used to characterize the vertical response of the tire and to evaluate the relationship between vertical load and the image-derived deformation metrics. The measured load–deflection characteristics are presented in Figure 15. As expected, vertical stiffness increased with inflation pressure, with approximate linear values of 175, 195, and 210 N/mm for 28, 32, and 36 psi inflation pressures, respectively. The relatively small difference between the 32 and 36 psi cases’ responses at low loads, followed by a clearer separation at higher loads, may reflect the combined contributions of carcass/sidewall compliance and inflation pressure to the vertical response.

**Figure 16:**
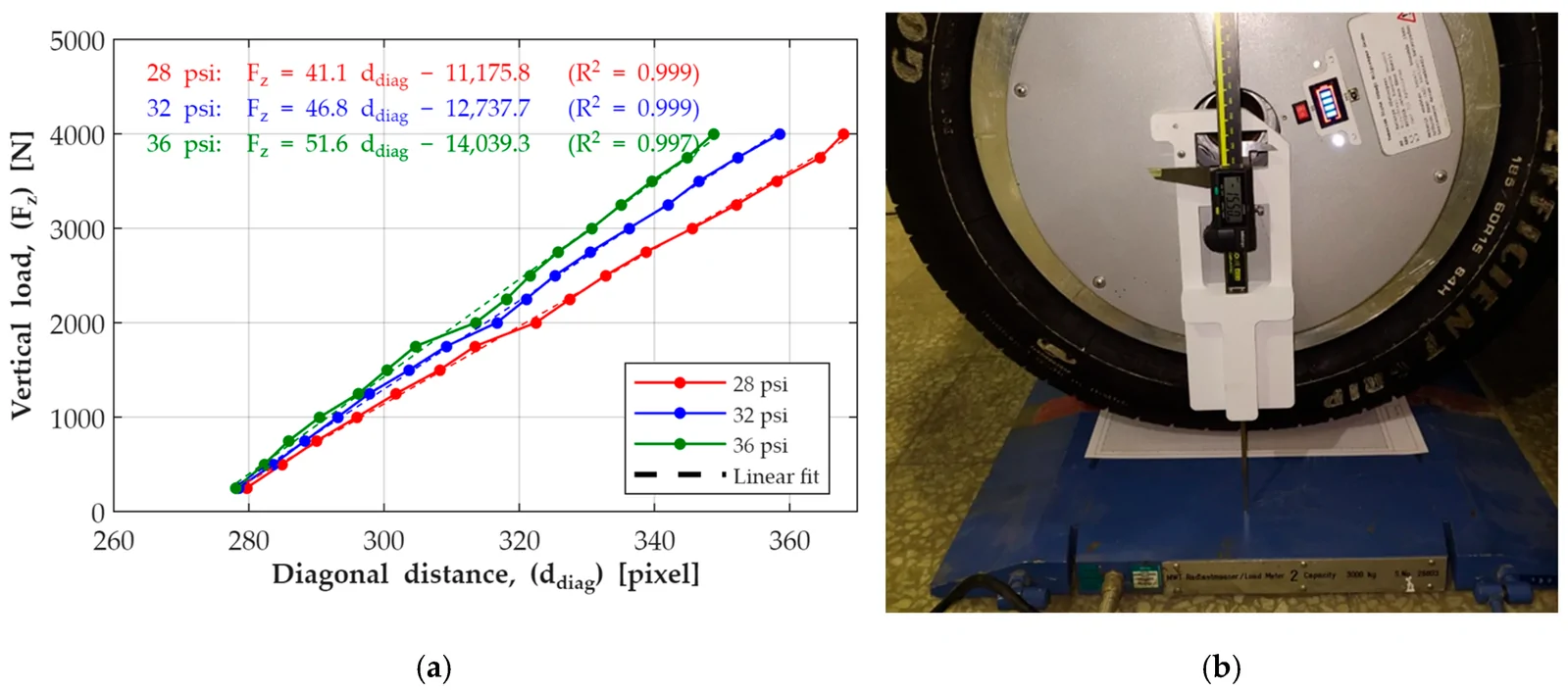
Image-based vertical-load estimation and vehicle-level verification: (**a**) Relationship between vertical load and the diagonal-distance metric obtained from the static tire tests. (**b**) Static-equilibrium measurement of the instrumented test vehicle using the MWT Load Meter wheel scale.

Among the evaluated image-derived quantities, the diagonal-distance metric exhibited the most consistent relationship with vertical load, as shown in Figure 16a. Since this metric is based on marker-center coordinates rather than the detected marker radii, it is less sensitive to local image blur, edge-detection uncertainty, and variations in apparent marker size. A local change in slope was observed around 1750 N; its origin could not be identified unambiguously, although the overall relationship remained monotonic over the investigated load range. These characteristics support the use of diagonal distance as an optical indicator of vertical wheel load. For the 28, 32, and 36 psi datasets, the pressure-specific linear fits yielded R^2^ values of 0.9989, 0.9992, and 0.9975 and RMSE values of 37.4, 33.4, and 58.1 N, respectively. The corresponding fitted sensitivities increased from 41.05 to 46.78 and 51.56 N pixel^−1^, demonstrating that the relationship was strongly linear but pressure-dependent.

To examine the applicability of the static calibration after installation on the vehicle, the smart wheel was mounted on the rear axle of the test vehicle and evaluated over multiple static wheel-load conditions, with the vehicle-installed configuration shown in Figure 16b. The 32 psi calibration obtained on the controlled tire-test system at 0° camber was used to estimate vertical wheel load from the measured diagonal-distance metric.

**Figure 17:**
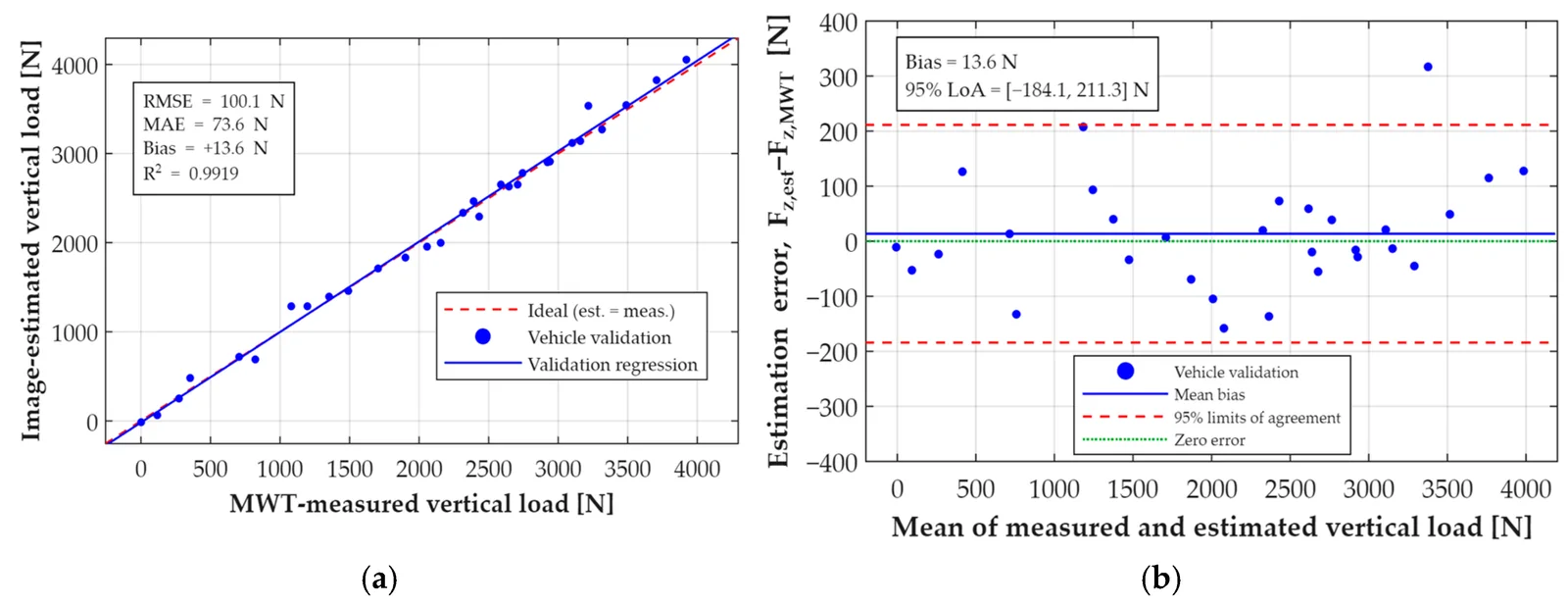
Vehicle-level multi-point validation of the image-based vertical-load estimation method: (**a**) Comparison of the image-estimated vertical wheel load with the reference load measured using the MWT Load Meter, including the ideal measured-equals-estimated line and linear regression. (**b**) Agreement plot showing the estimation error as a function of the mean measured and estimated vertical load, together with the mean bias, 95% limits of agreement, and zero-error reference.

Across all 30 vehicle-level validation conditions, covering reference wheel loads from approximately 0 to 3.92 kN, the optical estimates showed close agreement with the MWT Load Meter measurements. As shown in Figure 17a, the estimated vertical loads were in close agreement with the reference measurements and remained close to the 1:1 line. The mean bias was +13.6 N, while the mean absolute error (MAE) and root-mean-square error (RMSE) were 73.6 N and 100.1 N, respectively. The results yielded a coefficient of determination of R^2^ = 0.992. Linear regression between the estimated and measured loads gave F_z,est_ = 1.0147 F_z,MWT_ − 17.3 N, where F_z,est_ is the vertical load estimated using the image-derived calibration relationship, and F_z,MWT_ is the reference vertical load measured using the MWT wheel-load scale. The corresponding error distribution is shown in Figure 17b. The 95% limits of agreement were approximately −184.1 to +211.3 N, with no pronounced systematic increase in estimation error over the investigated load range, although the maximum absolute error reached 316.5 N. For the non-zero reference-load conditions, the mean absolute percentage error was 6.66%, with the largest relative deviations occurring at the lowest load levels, where comparatively small absolute errors result in large percentage errors. Since the vehicle-mounted wheel operated at approximately −1° to −2.5° camber during these measurements, the results indicate that the diagonal-distance calibration retained useful load-estimation capability after installation on the vehicle and under a small deviation from the calibration geometry, although camber sensitivity was not investigated independently.

**Figure 18:**
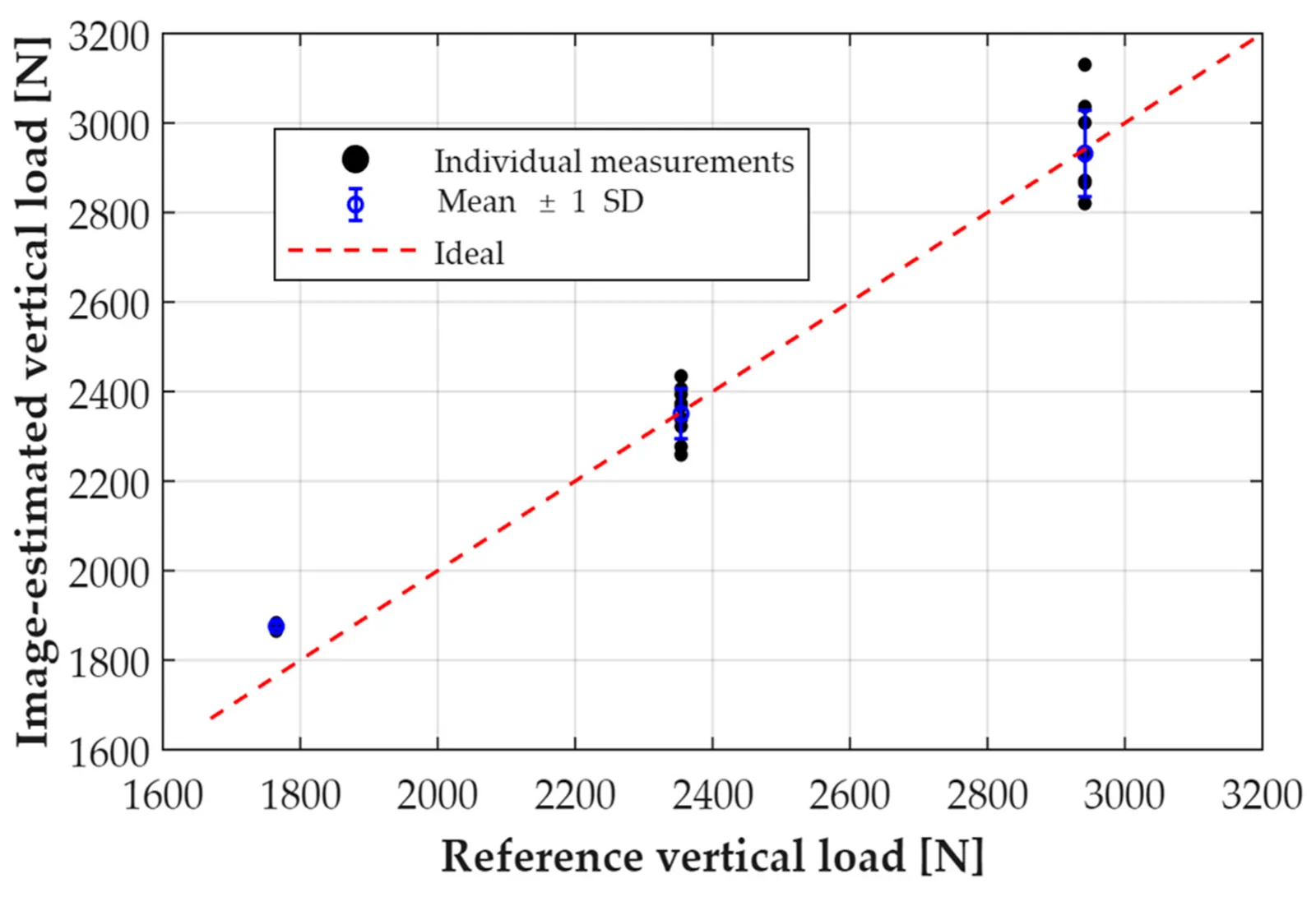
Repeatability of the image-based vertical-load estimation at three static reference load levels. Individual estimates from ten repetitions are shown together with the corresponding mean values and ±1 SD. The dashed line represents ideal agreement between the reference and image-estimated vertical loads.

The repeatability results are summarized in Figure 18. At the three reference load levels of 1765.2, 2353.6, and 2942.0 N (180, 240, and 300 kgf), the mean image-estimated vertical loads were 1875.9, 2351.0, and 2931.8 N, respectively. The corresponding within-condition standard deviations (SD) were 6.7, 55.6, and 96.7 N, yielding coefficients of variation of 0.36%, 2.36%, and 3.30%. Thus, the dispersion of the repeated estimates increased with vertical load, although the CV remained below 3.3% for all three conditions. The pooled within-condition SD over the complete repeatability dataset was 64.5 N. As shown in Figure 18, the lowest-load condition exhibited a highly concentrated cluster of repeated measurements but also a systematic positive offset, with a mean bias of +110.8 N. In contrast, the mean biases at the intermediate and highest load levels were only −2.6 and −10.2 N, respectively, despite their larger within-condition scatter. These results distinguish repeatability from absolute estimation accuracy and indicate that the image-based load estimate remained reproducible across independently re-established static loading conditions, with the greatest variability observed at the highest investigated load level.

### 3.2. Dynamic Tests

The dynamic measurements were evaluated using representative average-cycle responses obtained from the procedure described in Section 2.3. Because the cycle-selection procedure included an SD-based quality criterion, a representative average cycle could not be obtained for every individual test case. Therefore, the comparisons presented in this section were restricted to selected cases for which a sufficient number of consistent cycles remained after filtering. These cases were used to examine the effects of maneuver speed, inflation pressure, and turn direction on the measured tire-inner-liner deformation.

**Figure 19:**
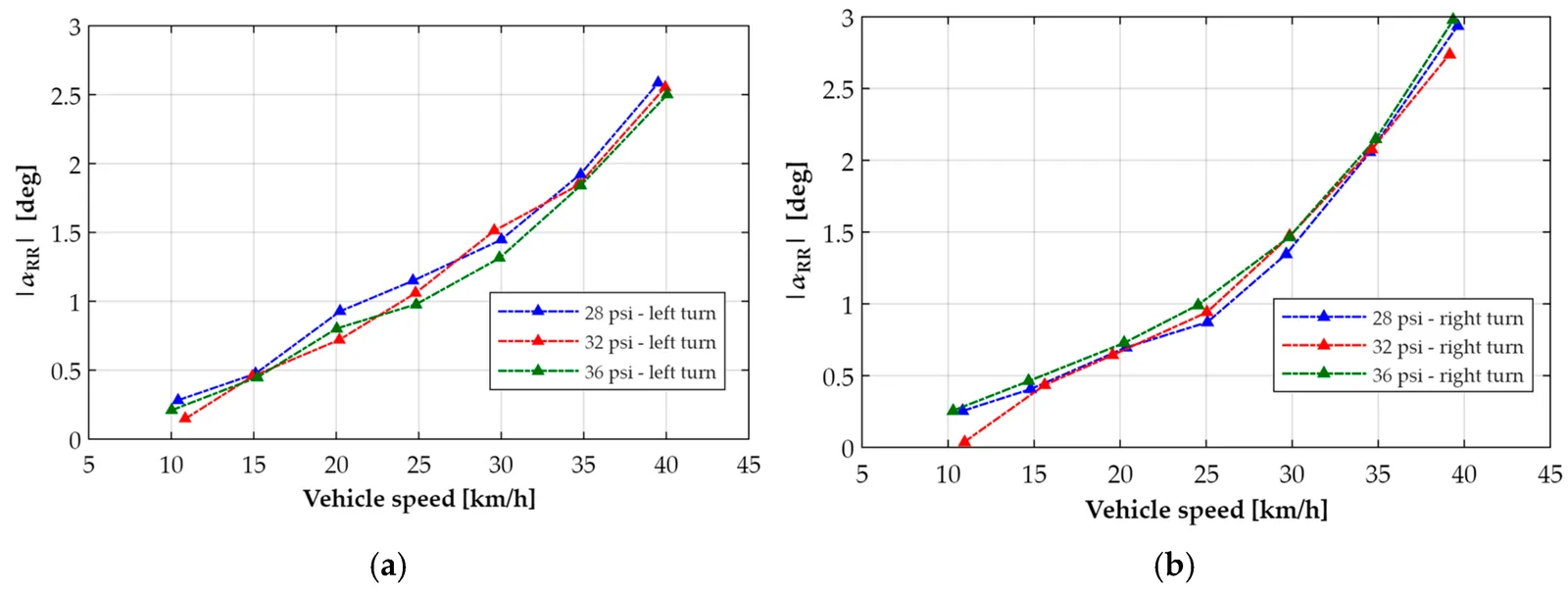
GPS-measured tire-slip angles (rear-right) during CRC tests for all investigated inflation pressures: (**a**) Left-turn maneuvers. (**b**) Right-turn maneuvers.

The vehicle speed and motion during the CRC maneuvers were independently recorded using the GPS measurement system. Figure 19a,b presents the measured slip angles α_RR_ at the instrumented rear-right wheel as a function of vehicle speed over the investigated range for the left- and right-turn maneuvers at all inflation pressures, respectively. These measurements are included primarily to document the actual operating conditions associated with the optical measurements.

#### 3.2.1. Effect of Maneuver Speed

**Figure 20:**
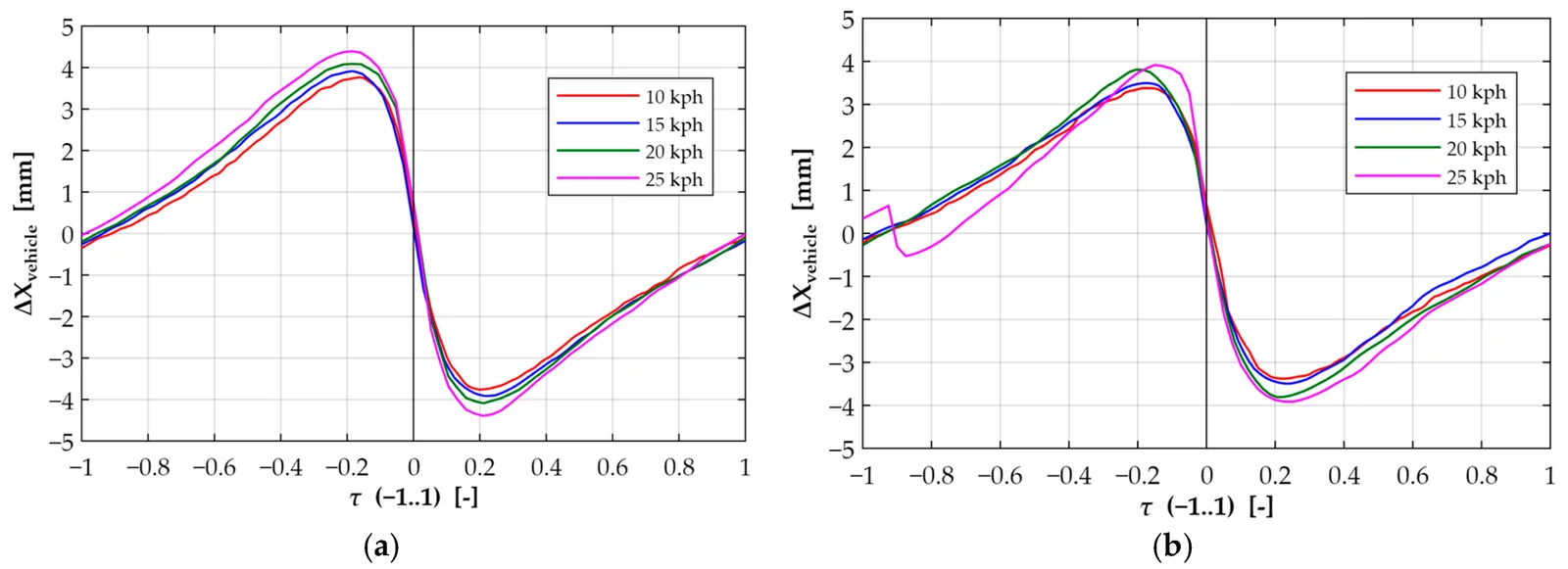
Effect of maneuver speed on longitudinal deformation at two inflation pressures: (**a**) 32 psi; (**b**) 36 psi.

The effect of maneuver speed on the measured longitudinal deformation is illustrated in Figure 20a,b for 32 and 36 psi inflation pressures, respectively.

For both inflation pressures, the amplitude of the longitudinal deformation increased with maneuver speed. The deformation amplitudes were also consistently higher at the lower inflation pressure. This trend is in parallel with the reduction in tire stiffness associated with decreasing inflation pressure, particularly in the vertical direction, which allows larger structural deformation under comparable loading conditions, while the influence of inflation pressure on lateral and longitudinal tire stiffness can also depend on the operating load and tire construction [36].

**Figure 21:**
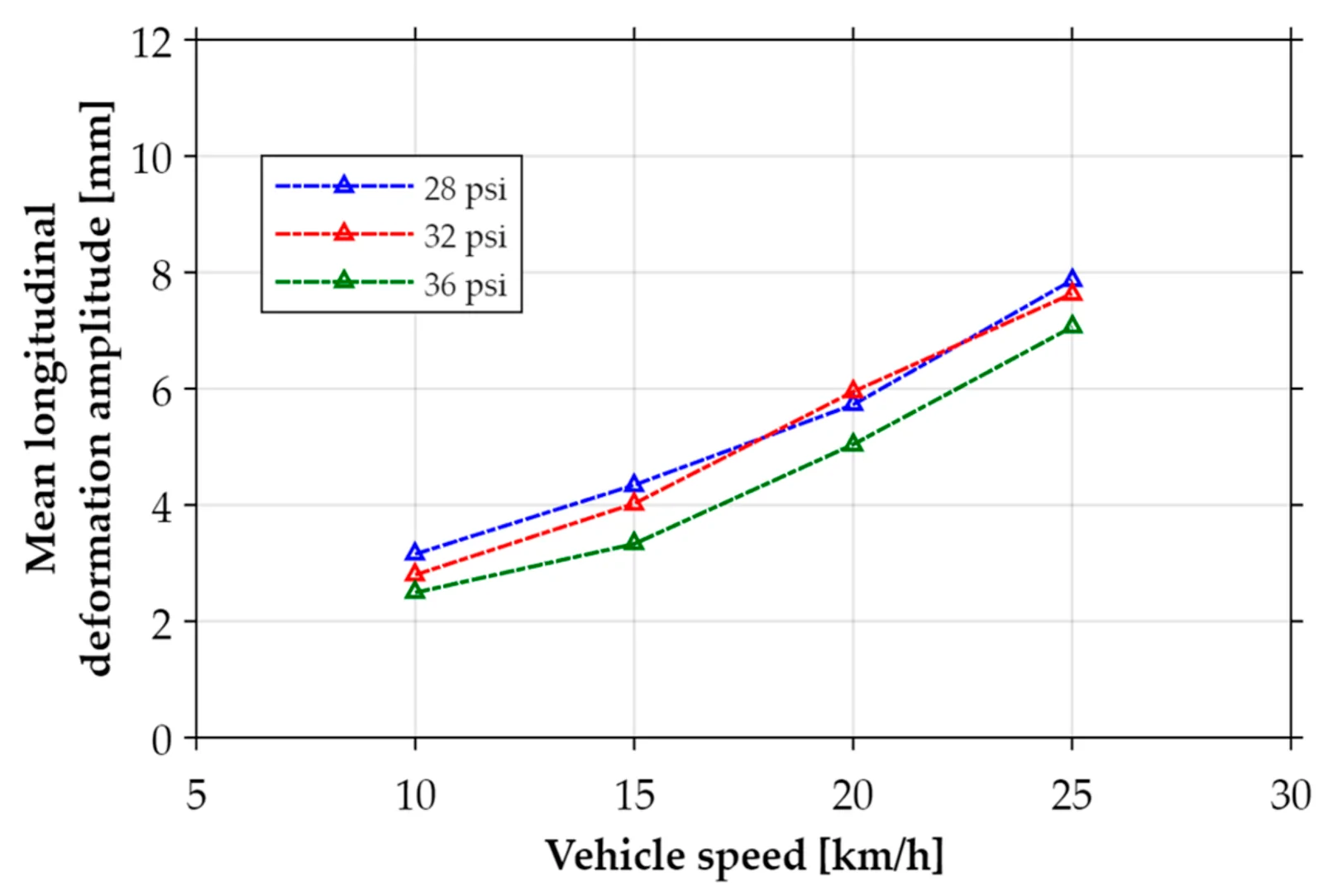
Mean longitudinal deformation amplitude as a function of maneuver speed for the investigated inflation pressures.

To summarize the overall trend, Figure 21 presents the mean longitudinal deformation amplitude as a function of maneuver speed for all investigated inflation pressures. Despite some overlap between the 28 and 32 psi cases, the results generally indicate increasing deformation amplitude with increasing maneuver speed and decreasing inflation pressure. The separation between the pressure levels is therefore not fully systematic over the entire test range. This may partly reflect the semi-controlled nature of the road tests, including variations in the actual maneuver speed, trajectory, and operating conditions. A more controlled test campaign with precisely regulated inflation pressure and maneuver speed would be required to quantify the pressure dependence more reliably.

**Figure 22:**
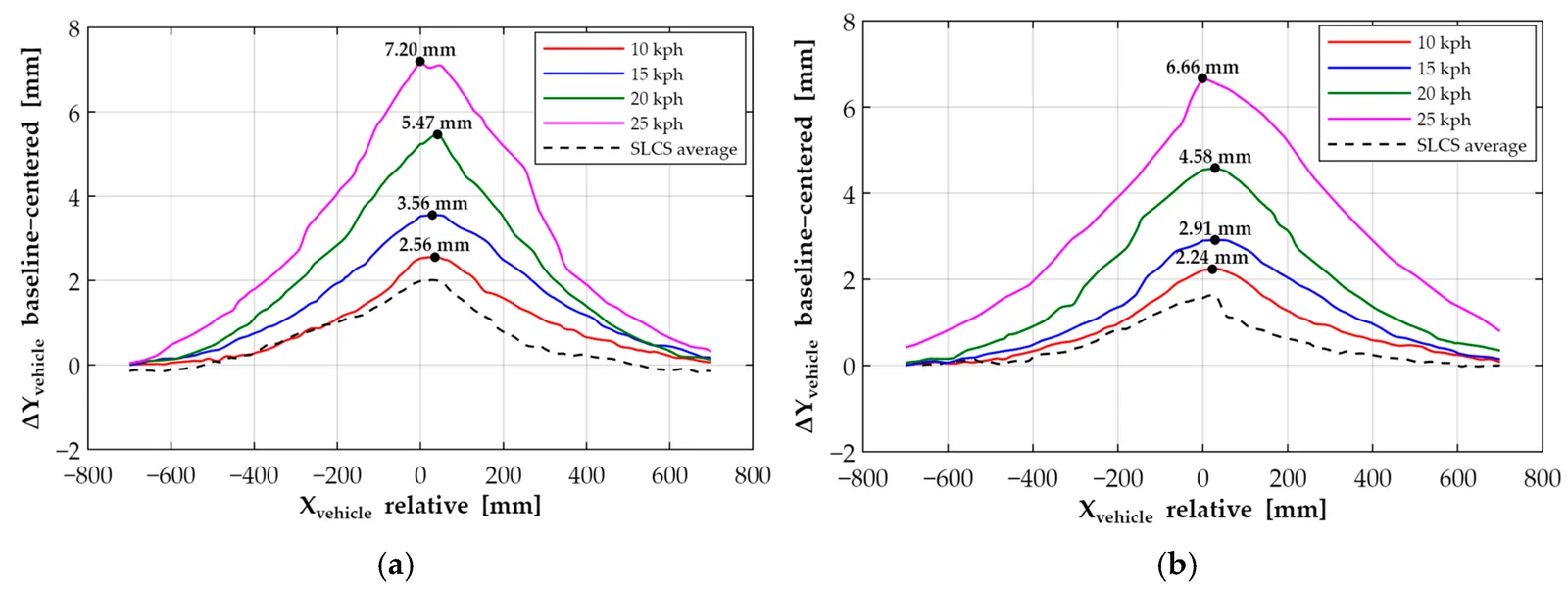
Effect of maneuver speed on lateral deformation at two inflation pressures: (**a**) 32 psi; (**b**) 36 psi. The black circles indicate the peak lateral-deformation values for each maneuver speed.

The lateral deformation responses are depicted in Figure 22a,b for 32 and 36 psi inflation pressures, respectively.

A similar trend was observed in the lateral deformation responses as in the longitudinal. As the maneuver speed increased, the magnitude of the lateral deformation increased for both inflation pressures. This behavior is consistent with the increase in lateral tire force required during higher-speed cornering. In addition, increasing the inflation pressure reduced the measured deformation, again reflecting the higher structural stiffness of the tire at increased inflation pressure.

**Figure 23:**
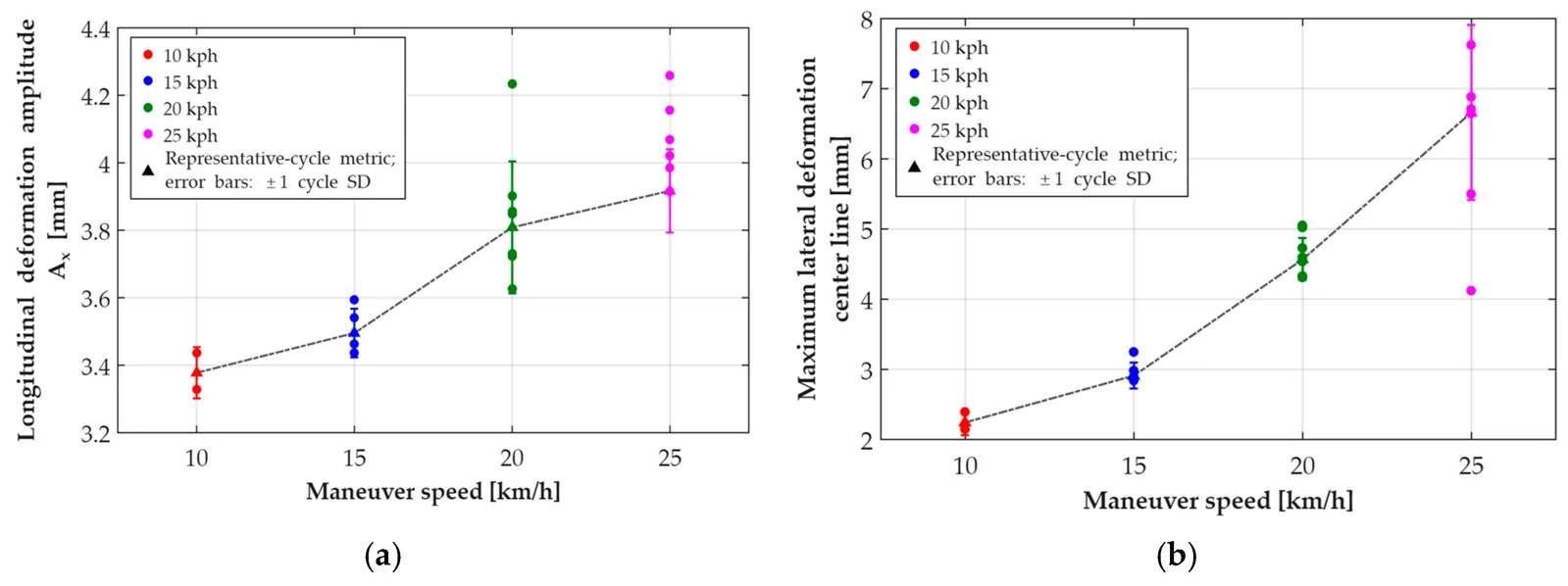
Cycle-to-cycle variation in (**a**) longitudinal center-line amplitude and (**b**) lateral center-line peak at different maneuver speeds during 20 m radius left-turn CRC tests at 36 psi. Circles show individual-cycle values; triangles and error bars indicate the representative-cycle metric and ±1 cycle SD, respectively.

Figure 23a,b presents the effect of maneuver speed on the longitudinal and lateral deformation metrics during the 20 m radius left-turn CRC maneuver at 36 psi, respectively. A total of 19 complete cycles were obtained across the four speeds, with 2, 4, 7, and 6 cycles available at 10, 15, 20, and 25 km/h, respectively. All candidate cycles passed the quality criteria, and no cycle was rejected. Nevertheless, the variability estimate at 10 km/h should be interpreted cautiously because it is based on only two complete cycles.

The longitudinal center-line amplitude extracted from the representative cycle increased monotonically from 3.378 mm at 10 km/h to 3.495, 3.809, and 3.917 mm at 15, 20, and 25 km/h, respectively. The overall increase between 10 and 25 km/h was 0.539 mm, corresponding to approximately 16.0%. The SD of the individual-cycle amplitudes remained between 0.073 and 0.196 mm, with coefficients of variation ranging from 2.07% to 5.10%. The pooled within-case SD was 0.148 mm, whereas the between-speed SD of the representative-cycle amplitudes was 0.255 mm, giving a between-to-within standard-deviation ratio of 1.72. The range of the representative-cycle amplitudes was 3.64 times the pooled within-case SD, indicating that the observed longitudinal increase with speed was larger than the typical cycle-to-cycle dispersion, although relatively modest in absolute magnitude.

A substantially stronger speed dependence was observed in the lateral response. The representative-cycle center-line peak increased from 2.243 mm at 10 km/h to 2.911 mm at 15 km/h, 4.568 mm at 20 km/h, and 6.660 mm at 25 km/h. Thus, the lateral peak increased by 4.417 mm between 10 and 25 km/h, corresponding to approximately 197%, or nearly three times its value at 10 km/h. The single-cycle SD remained between 0.178 and 0.299 mm up to 20 km/h but increased markedly to 1.248 mm at 25 km/h. Correspondingly, the CV increased from 6.43% at 20 km/h to 19.98% at 25 km/h, indicating increased cycle-to-cycle variability at the highest investigated speed.

For the lateral response, the between-speed SD of the representative-cycle peaks was 1.969 mm, compared with a pooled within-case SD of 0.751 mm. The resulting between-to-within SD ratio was 2.62, while the total range of the representative-cycle peaks was 5.88 times the pooled within-case SD. These descriptive comparisons show that the change in lateral deformation between maneuver speeds was appreciably larger than the typical variation between repeated cycles at the same speed. The particularly large dispersion at 25 km/h, however, indicates that the repeatability of the lateral peak decreased as the maneuver severity increased.

**Figure 24:**
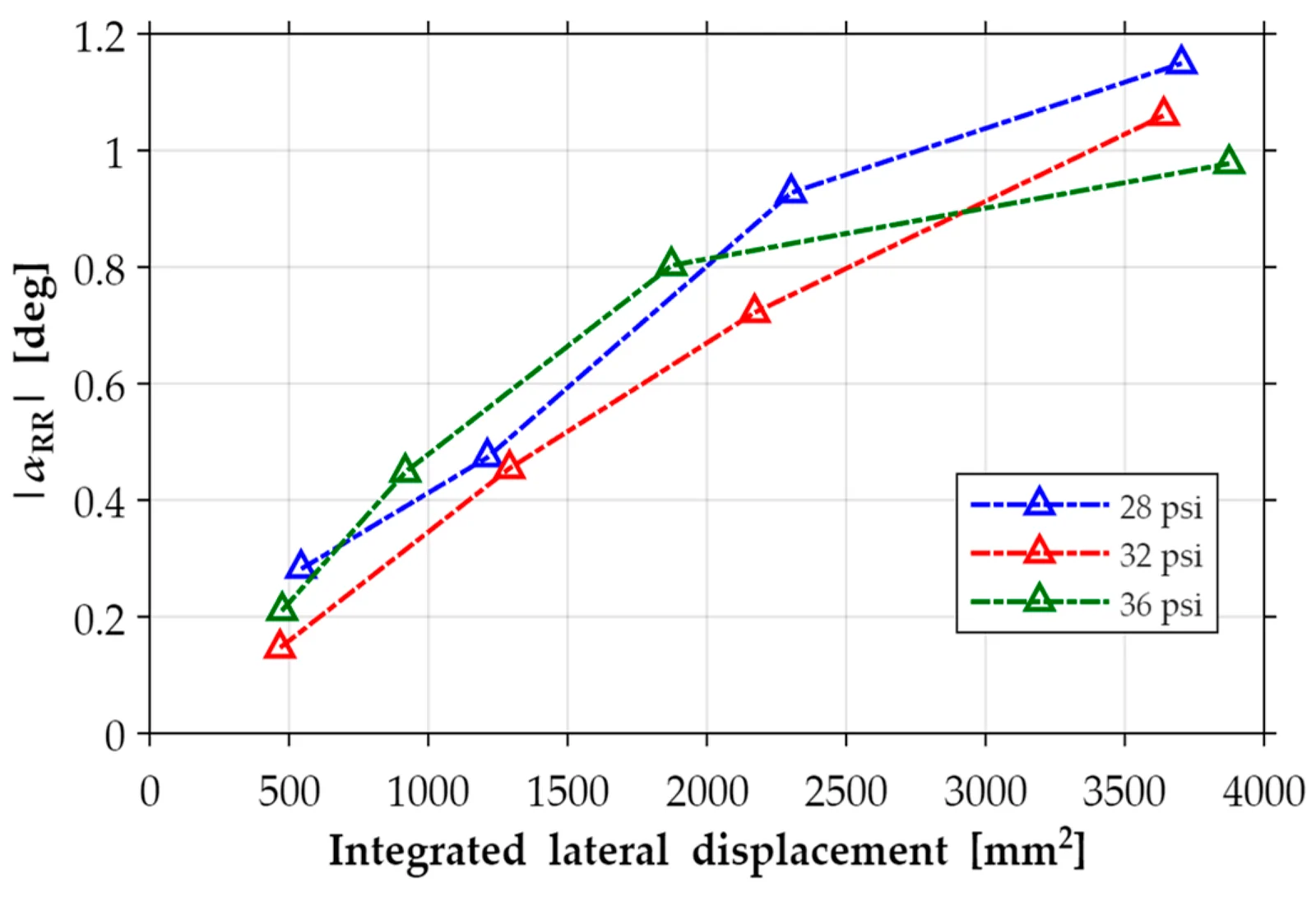
Relationship between the slip angle and the area enclosed between the CRC lateral-deformation response and the corresponding SLCS reference.

In addition to the peak deformation values, the area enclosed between the CRC lateral-deformation response and the corresponding SLCS reference was considered as an integrated measure of the overall lateral deformation of the tire over one wheel cycle. As shown in Figure 24, this area-based metric exhibits an overall increasing tendency with the measured slip angle, although the relationship is not strictly linear. Since the metric reflects the deformation response over both the contact and non-contact portions of the tire, it may provide information related to the overall lateral loading of the tire rather than to a single local deformation value. For small slip angles, lateral tire force is approximately proportional to slip angle, with the corresponding slope defined as the cornering stiffness. However, the instrumented right-rear wheel also operated with a non-zero static camber angle; therefore, the measured lateral response may include the combined effects of slip angle and camber-induced lateral force [37]. Consequently, the absence of a clear linear relationship in Figure 24 does not necessarily rule out a relationship between the proposed deformation metric and lateral tire force. More controlled and repeated experiments, particularly with controlled slip angle and camber conditions, would be required to evaluate this relationship quantitatively.

#### 3.2.2. Effect of Inflation Pressure

**Figure 25:**
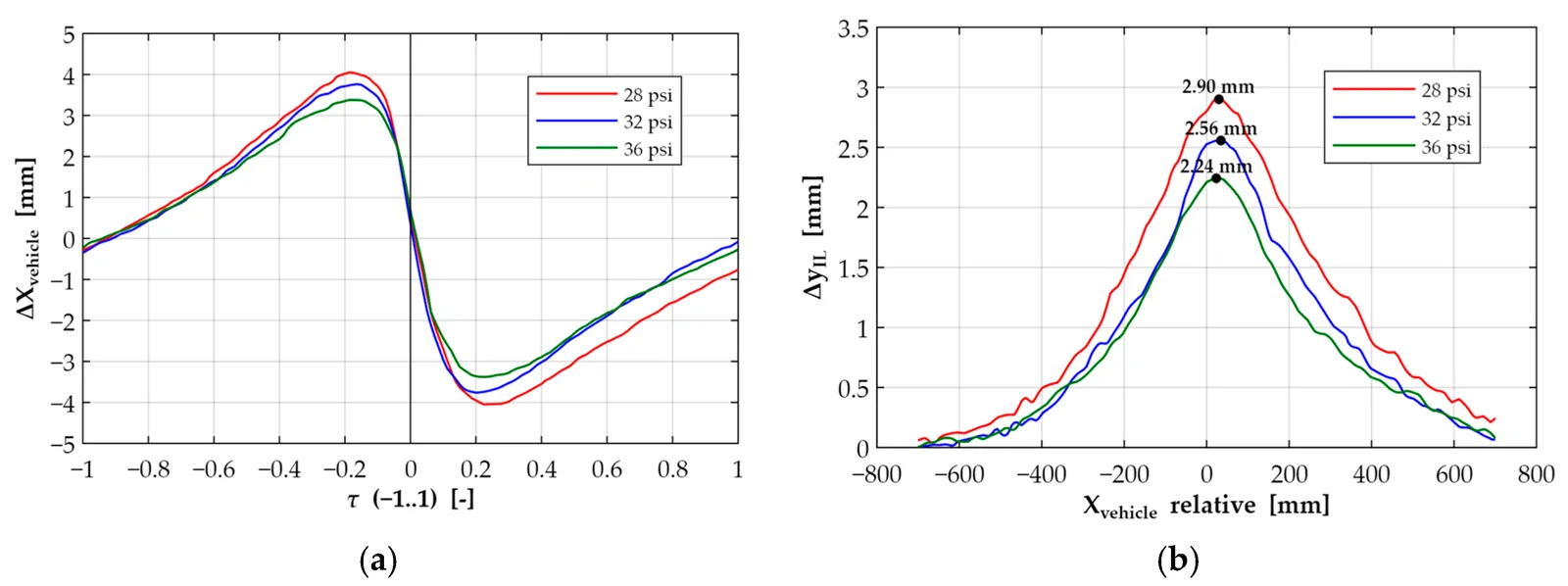
Effect of inflation pressure on tire-inner-liner deformation during a 10 kph left-turn CRC maneuver: (**a**) Longitudinal deformation. (**b**) Lateral deformation. The black circles indicate the peak lateral-deformation values for each pressure condition.

The influence of inflation pressure is shown more directly in Figure 25, where the deformation responses obtained at the investigated pressure levels are compared for the same maneuver condition. The effect is evident in both the longitudinal and lateral responses: increasing inflation pressure results in a reduction in deformation magnitude. Although this tendency is already visible in the speed-dependent comparisons, Figure 25a,b demonstrates the pressure dependence more clearly by comparing all investigated inflation pressures under the same operating condition, with longitudinal and lateral deformations, respectively.

Although the pressure-dependent deformation analysis was conducted at 10 km/h, the 25 km/h cases were selected for the cycle-to-cycle variability assessment in because they provided 6–9 complete cycles per pressure, compared with only 2–3 cycles at 10 km/h. A total of 23 cycles were evaluated at 25 km/h, comprising 8, 9, and 6 cycles at 28, 32, and 36 psi, respectively. All candidate cycles satisfied the quality criteria, and none was rejected.

**Figure 26:**
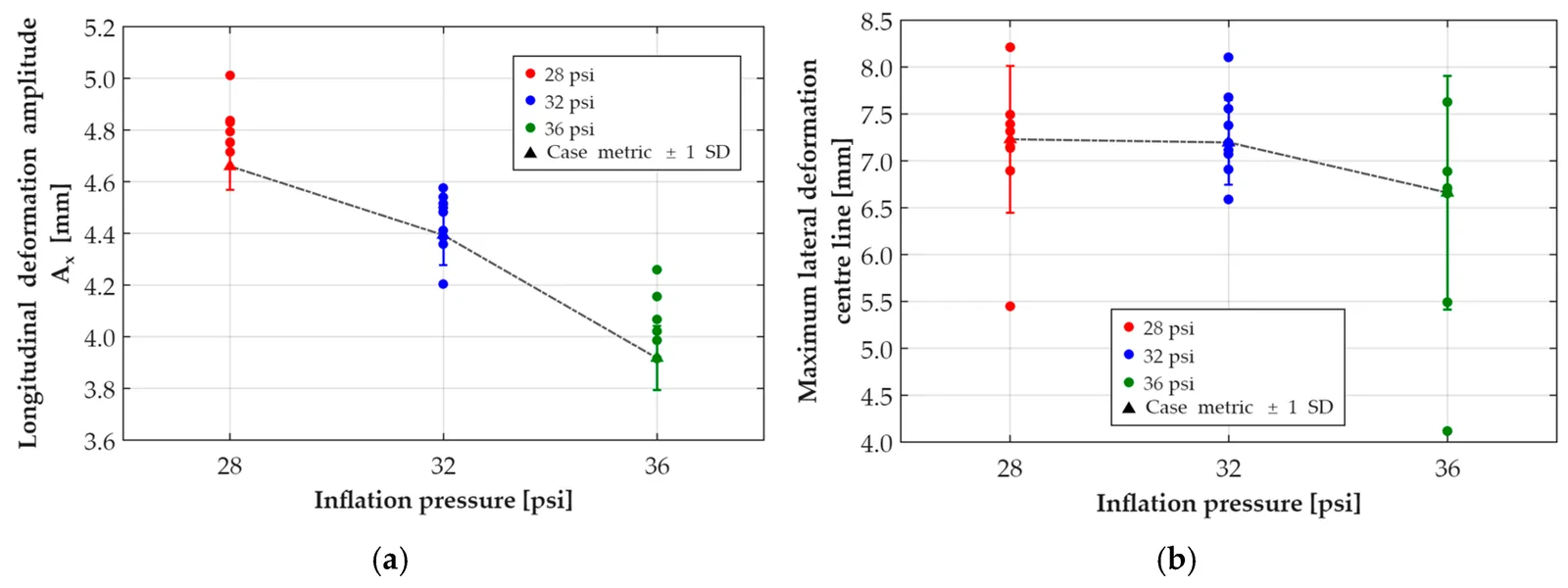
Cycle-to-cycle variation in (**a**) longitudinal center-line amplitude and (**b**) lateral center-line peak at different inflation pressures during 25 km/h, 20 m radius left-turn CRC tests. Circles show individual-cycle values; triangles and error bars indicate the representative-cycle metric and ±1 cycle SD, respectively.

As shown in Figure 26a, the representative-cycle longitudinal amplitude decreased monotonically from 4.660 mm at 28 psi to 4.393 mm at 32 psi and 3.917 mm at 36 psi. The total reduction was 0.743 mm, corresponding to 15.9%. The single-cycle SD values were relatively small at 0.091, 0.115, and 0.124 mm, with respective coefficients of variation of 1.88%, 2.59%, and 3.05%. The between-pressure SD of the representative-cycle amplitudes was 0.376 mm, approximately 3.44 times the pooled within-case SD of 0.110 mm. Thus, the pressure-dependent change in longitudinal amplitude was clearly larger than the typical cycle-to-cycle dispersion.

The lateral center-line peak exhibited a weaker pressure dependence, as presented in Figure 26b. The representative-cycle peak changed only slightly from 7.230 mm at 28 psi to 7.196 mm at 32 psi before decreasing to 6.660 mm at 36 psi. The overall reduction from 28 to 36 psi was 0.570 mm, or 7.9%. In contrast to the longitudinal response, the lateral cycle-to-cycle dispersion was substantial: the SD values were 0.783, 0.450, and 1.248 mm, with coefficients of variation of 10.97%, 6.17%, and 19.98%, respectively. The pooled within-case SD of 0.827 mm exceeded the between-pressure SD of 0.320 mm. Consequently, the observed pressure-related variation in the lateral peak was smaller than the cycle-to-cycle variability, particularly at 36 psi.

#### 3.2.3. Effect of Maneuver Direction

**Figure 27:**
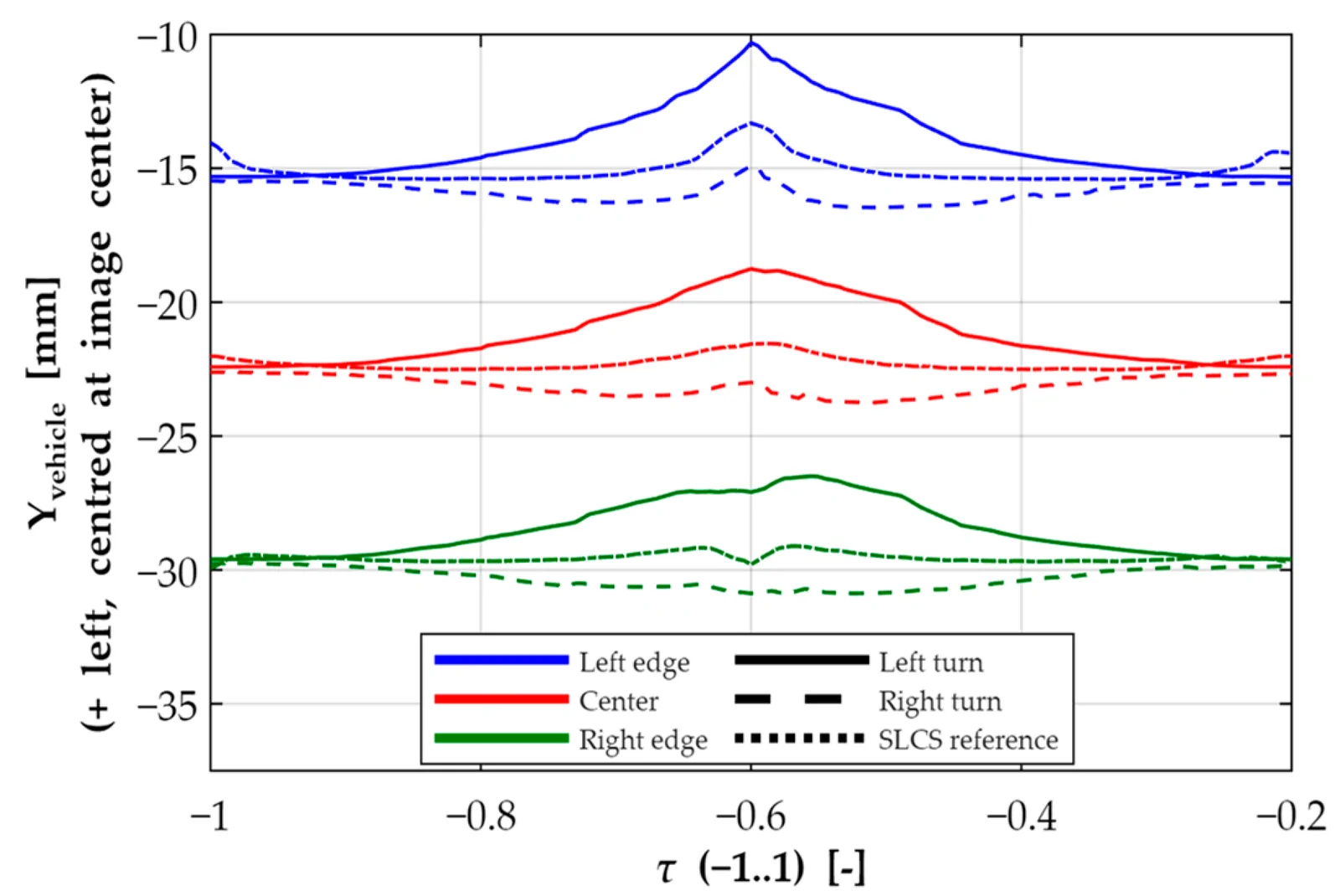
Effect of turn direction on lateral deformation at 30 kph and 32 psi inflation pressure.

The effect of turn direction on the lateral deformation response is presented in Figure 27. Relative to the corresponding SLCS reference, the deformation response is asymmetric between left- and right-turn maneuvers, as expected from the different vertical and lateral loading conditions of the instrumented rear-right wheel during maneuver.

For the selected condition, both the peak difference from the SLCS reference and the area enclosed between the CRC and reference responses were greater during the left-turn maneuver than during the corresponding right-turn maneuver. During a left turn, lateral load transfer increases the vertical load carried by the right-side wheels. Consequently, the instrumented right-rear tire carries a larger share of the rear-axle cornering force, resulting in a greater lateral deformation response. During a right turn, the opposite load-transfer tendency reduces the vertical load acting on the instrumented wheel. Consequently, its contribution to the rear-axle cornering force decreases, leading to a lower lateral deformation response.

## 4. Discussion

The static vertical-load assessment enabled the performance of the diagonal-distance metric to be quantified against reference load measurements. The pressure-specific linear relationships obtained at 28, 32, and 36 psi yielded R^2^ values of 0.9989–0.9992 and RMSE values of 33.4–58.1 N. The 32 psi static diagonal-distance data were subsequently used for vehicle-level multi-point validation over 30 static conditions and loads up to approximately 3.92 kN. This assessment yielded a prediction R^2^ of 0.992, an RMSE of 100.1 N, an MAE of 73.6 N, and a mean bias of +13.6 N. These findings provide an initial quantitative validation of the diagonal-distance metric for static vertical-load measurement under the tested configuration, while the dynamic force- and slip-angle-related responses are interpreted in terms of observed sensitivities and potential associations.

The trends observed during the dynamic road tests were physically consistent with established tire behavior. The reduction in deformation with increasing inflation pressure agrees with the increase in tire structural stiffness reported in conventional tire-mechanics studies [37]. Likewise, the increase in lateral deformation with maneuver speed and the asymmetry between left- and right-turn maneuvers are consistent with corresponding changes in lateral tire force and wheel-load transfer. However, the proposed metrics are not uniquely sensitive to individual physical quantities. The diagonal-distance metric is affected by vertical load, inflation pressure, and contact geometry; longitudinal amplitude can be influenced by tire stiffness, vertical loading, rolling conditions, and longitudinal slip; and the lateral peak can be influenced by lateral force, slip angle, wheel-load transfer, maneuver direction, and road-surface disturbances. The dynamic results should therefore be interpreted as sensitivity to combined operating conditions rather than independent estimates of force or slip angle.

An important aspect of the present study is that these trends were obtained under on-road rather than highly controlled laboratory conditions. During the dynamic tests, the prescribed trajectory and vehicle speed could not be reproduced perfectly, and the measurements were also affected by road-surface irregularities and other uncontrolled disturbances. The number of retained cycles varied from 2 to 9 among the evaluated cases. The longitudinal metric exhibited cycle-to-cycle coefficients of variation of 1.88–5.10%, whereas the corresponding lateral values were 6.08–19.98%, indicating greater cycle-to-cycle variability in the lateral response. For the dynamic analysis, the current applicability of the method is consequently limited to comparative deformation measurements for the tested tire–vehicle configuration and operating ranges. Transfer to different tires, marker patterns, camera positions, inflation-pressure ranges, or more severe transient maneuvers requires additional calibration and validation. Moreover, application requires the marker grid to remain visible and reliably detectable throughout the relevant part of the wheel rotation. Controlled dynamic testing with synchronized reference measurements would enable quantitative relationships to be established between the optical metrics and tire–vehicle states, thereby providing a calibration basis for subsequent estimation of kinematic and dynamic parameters during on-road operation.

Potential measurement errors arise from residual camera-calibration error, local tire curvature and out-of-plane motion, marker-application and centering errors, illumination variation, motion blur, partial occlusion, and occasional marker-grid misidentification. The distance-dependent imaging assessment and local pixel-to-length conversion reduce sensitivity to changes in apparent marker size, while the cycle-quality criteria limit the influence of non-physical detection jumps. Nevertheless, these procedures cannot eliminate all perspective-, scaling-, and detection-related uncertainty.

Compared with more elaborate optical approaches based on stereo digital image correlation, which aim to reconstruct the full three-dimensional deformation field of the tire inner liner [25], the present method intentionally adopts a simpler measurement philosophy. Rather than reconstructing the complete deformation field, a limited set of geometric features is extracted from the marker grid and interpreted in relation to tire loading and deformation. This distinction is important for applications in which physically interpretable tire-state information is required without the hardware and computational complexity associated with full-field stereo-based measurements.

## 5. Conclusions

A single-camera optical smart-wheel prototype was developed to measure tire inner-liner deformation using a circular-marker grid and physically interpretable image-based metrics. Static tests demonstrated a strong, inflation-pressure-dependent relationship between the diagonal-distance metric and vertical wheel load, while subsequent vehicle-level multi-point validation showed close agreement with reference wheel-load measurements. Dynamic road tests showed increasing longitudinal and lateral deformation with increasing maneuver speed and decreasing inflation pressure, together with direction-dependent lateral responses consistent with wheel-load transfer. Cycle-to-cycle analysis indicated comparatively repeatable longitudinal responses and greater variability in the lateral response under some operating conditions. The dynamic results demonstrate the sensitivity of the proposed metrics to changes in tire operating conditions and indicate a potential association between lateral deformation and tire slip angle; however, they should not be interpreted as validated tire-force or slip-angle estimates. Overall, the findings support the feasibility of a lower-complexity optical approach that avoids full-field stereo-based three-dimensional reconstruction. Future work should establish quantitative metric–state relationships under controlled dynamic conditions using synchronized wheel-force and kinematic reference measurements, assess their transferability to on-road state estimation, and address higher marker-grid resolution, real-time onboard processing, miniaturization, and energy harvesting toward a compact self-contained smart-wheel system.

## Figures and Tables

**Table 1:** Intrinsic and extrinsic camera calibration results obtained using the MATLAB Camera Calibrator.

Parameter	Symbol	Value	Unit
Horizontal focal length	$f_{x}$	1168.71	pixels
Vertical focal length	$f_{y}$	1173.82	pixels
Principal point, horizontal coordinate	$c_{x}$	769.87	pixels
Principal point, vertical coordinate	$c_{y}$	606.79	pixels
First radial distortion coefficient	$k_{1}$	0.01969	-
Second radial distortion coefficient	$k_{2}$	−0.03369	-
Mean rotation-vector standard-error norm	-	0.00122	rad
Mean translation-vector standard-error norm	-	0.146	mm
Mean reprojection error	-	0.759	pixels

## Data Availability

The raw image dataset generated in this study, comprising approximately 18,000 BMP images, is not publicly available because it forms part of an ongoing research program and is intended to support further publications. Compressed MP4 video sequences derived from the 42 test cases are provided as Supplementary Materials for visual reference; however, these videos do not constitute a substitute for the original image data used in the analysis. The GPS datasets and the image-processing and analysis codes are not publicly archived at this stage. Further inquiries regarding data supporting the results reported in this article may be directed to the corresponding author.

## Supplementary Materials

The following supporting information can be downloaded at https://www.mdpi.com/article/10.3390/s26196284/s1. Videos S1–S42: Rim-mounted camera recordings of the tire inner liner during constant-radius cornering (CRC) tests under different maneuver speeds, turning radii, inflation pressures, and turning directions. The video filenames follow the format CRC_[speed]kph_[radius]m_[pressure]psi_[direction].mp4, where speed denotes the nominal maneuver speed, radius the nominal turning radius, pressure the tire inflation pressure, and direction the turning direction (left or right).

## Abbreviations

The following abbreviations are used in this manuscript:

BMP	Bitmap image file
CHT	Circular Hough Transform
CRC	Constant-radius cornering
CV	Coefficient of variation
DIC	Digital image correlation
GPS	Global positioning system
LED	Light-emitting diode
MAE	Mean absolute error
PLA	Polylactic acid
RMSE	Root mean square error
SD	Standard deviation
SLCS	Straight-line constant-speed
VTT	Technical Research Centre of Finland
